# Microbial succession and assembly shaped by sulfur, spatial partitioning, and water flow in a volcanic acidic river of northern Patagonia

**DOI:** 10.1093/ismejo/wrag048

**Published:** 2026-03-09

**Authors:** Duarte-Ramírez Juan, Arisan Dilanaz, Rojas-Villalobos Camila, Díaz-González Fernando, Sepúlveda-Rebolledo Pedro, Moya-Beltrán Ana, Ulloa Ricardo, Johnson D Barrie, Vera Mario, Beatriz Díez, Castro Matías, Temporetti Pedro, Giaveno Alejandra, Issotta Francisco, Quatrini Raquel

**Affiliations:** Centro Científico y Tecnológico de Excelencia Ciencia & Vida, Santiago 8580702, Chile; Programa de Doctorado en Biotecnología y Bioemprendimiento, Facultad de Medicina, Universidad San Sebastián, Santiago 8420524, Chile; Centro Científico y Tecnológico de Excelencia Ciencia & Vida, Santiago 8580702, Chile; Programa de Doctorado en Biotecnología y Bioemprendimiento, Facultad de Medicina, Universidad San Sebastián, Santiago 8420524, Chile; Centro Científico y Tecnológico de Excelencia Ciencia & Vida, Santiago 8580702, Chile; Programa de Doctorado en Biología Computacional, Facultad de Ingeniería, Arquitectura y Diseño, Universidad San Sebastián, Santiago 8420524, Chile; Centro Científico y Tecnológico de Excelencia Ciencia & Vida, Santiago 8580702, Chile; Programa de Doctorado en Biotecnología y Bioemprendimiento, Facultad de Medicina, Universidad San Sebastián, Santiago 8420524, Chile; Centro Científico y Tecnológico de Excelencia Ciencia & Vida, Santiago 8580702, Chile; Departamento de Informática y Computación, Facultad de Ingeniería, Universidad Tecnológica Metropolitana, Santiago 7800002, Chile; PROBIEN (CCT Patagonia Confluencia-CONICET, UNCo), Facultad de Ingeniería, Departamento de Química, Universidad Nacional del Comahue, Neuquén Q8300, Argentina; School of Environmental and Natural Sciences, Bangor University, Bangor LL57 2UW, United Kingdom; Faculty of Health and Life Sciences, Coventry University, West Midlands, Coventry CV1 5FB, United Kingdom; Natural History Museum, London SW7 5BD, United Kingdom; Instituto de Ingeniería Biológica y Médica, Escuelas de Ingeniería, Medicina y Ciencias Biológicas, Pontificia Universidad Católica de Chile, Macul, Santiago 7820436, Chile; Departamento de Ingeniería de Minería, Escuela de Ingeniería, Pontificia Universidad Católica de Chile, Macul, Santiago 7820436, Chile; Centro Científico y Tecnológico de Excelencia Ciencia & Vida, Santiago 8580702, Chile; Centro GEMA - Genómica, Ecología & Medio Ambiente, Universidad Mayor, Santiago 8580745, Chile; Millennium Institute Center for Genome Regulation (CGR), Santiago 8320165, Chile; Instituto Milenio de Oceanografía (IMO), Universidad de Concepción, Concepción 4070386, Chile; Instituto de Investigaciones en Biodiversidad y Medioambiente (INIBIOMA), Centro Regional Universitario Bariloche-UNComahue, CCT-Patagonia Norte, CONICET, San Carlos de Bariloche 8400, Argentina; PROBIEN (CCT Patagonia Confluencia-CONICET, UNCo), Facultad de Ingeniería, Departamento de Química, Universidad Nacional del Comahue, Neuquén Q8300, Argentina; Centro Científico y Tecnológico de Excelencia Ciencia & Vida, Santiago 8580702, Chile; Centro GEMA - Genómica, Ecología & Medio Ambiente, Universidad Mayor, Santiago 8580745, Chile; Centro Científico y Tecnológico de Excelencia Ciencia & Vida, Santiago 8580702, Chile; Facultad de Ciencias, Universidad San Sebastián, Santiago 8420524, Chile

**Keywords:** acidophiles, deterministic processes, stochastic processes, dispersal limitation, water flow, sulfur cycling, sulfur bead, microhabitat, free-living, particle-attached

## Abstract

Extreme acidic environments represent natural laboratories for investigating the mechanisms of microbial community assembly, yet the ecological processes structuring these communities remain incompletely understood. Here, we investigate how spatial partitioning, hydrodynamics, and colonization history shape microbial succession in a unique sulfur-rich, acidic river of volcanic origin in northern Patagonia. We combined 16S rRNA gene profiling and shotgun metagenomics with a multi-scale experimental framework encompassing water column fractionation and colonization assays under native and controlled conditions. Microbial diversity was strongly influenced by spatial fractionation, with free-living communities exhibiting higher richness and temporal variability than particle-associated assemblages. Water flow modulated community structure, increasing evenness in free-living fractions under high-flow conditions, but had limited impact on particle-attached communities. Colonization of sulfur-beads followed a structured successional trajectory, with autotrophic sulfur oxidizers dominating early stages and heterotrophs adapted to biofilm lifestyles increasing over time. *Ex situ* recolonization assays revealed strong priority effects, with initial colonizers determining successional trajectories. Turnover analyses revealed that the balance among stochastic and deterministic assembly processes shifted across communities with pronounced stochasticity in the water column and flow-dependent effects in free-living communities, while biofilm associated communities on sulfur-beads exhibited stronger contribution of deterministic selection. These ecological patterns were mirrored by functional differentiation, with gene enrichment analyses revealing adaptive signatures of substrate attachment and resource acquisition. By integrating fine-scale environmental variation with colonization dynamics, this study reveals how microscale habitat structure and temporal fluxes jointly modulate microbial community assembly rules, offering a nuanced framework to dissect ecological processes in extreme systems.

## Introduction

Understanding how microbial communities assemble and persist under diverse environmental conditions remains a fundamental challenge in microbial ecology [1]. While significant progress has been made in disentangling the roles of selection, dispersal, drift, and historical contingency in community assembly [2, 3], the interplay between these processes is often context-dependent, and remains poorly resolved across ecosystems. Extremely acidic environments, defined by pH values below 3, support highly specialized, low-to-moderate diversity microbial communities [4–6], which makes them particularly amenable to species-level resolution studies. This has been effectively demonstrated in acid mine drainages (AMDs) systems such as Iron Mountain in the US [7, 8], Rio Tinto in the Iberian Pyritic Belt [9, 10], and the Mynydd Parys prehistoric copper mine in Whales [11, 12]. Compared to the extensively studied AMD environments, the microbial ecology of natural acidic systems has received far less attention, excepting well-characterized sulfidic cave systems (e.g. [6, 13]) and volcanically influenced sites (e.g. Yellowstone [14, 15], Dallol–Danakil [16], Taupō [17]). Accumulating evidence suggest that current AMD-based knowledge on microbial succession and assembly dynamics (e.g. [18–20]), does not fully capture the processes occurring in volcanic acidic rivers, which differ in their origin, complexity of chemical gradients, and longevity, and support greater taxonomic, ecophysiological, and evolutionary diversification than mine sites (e.g. [21]).

The Copahue-Caviahue Volcanic Complex (CCVC) in northern Patagonia [22], features the Río Agrio Superior (RAS), an acidic river whose headwaters are fed by geothermal waters from Copahue volcano’s hot springs. Its flow and chemistry are modulated by snowmelt and tributary inflows [23], which create steep physicochemical gradients that support highly specialized microbial communities [24–28]. High elemental sulfur accumulation rates across RAS sites, driven primarily by sulfur dioxide disproportionation [29], structure microbial habitats and support sulfur-cycling assemblages dominated by *Acidithiobacillia* [25, 26].

The *Acidithiobacillia* are among the most globally widespread and ecologically significant acidophiles involved in inorganic sulfur compounds transformations under extreme acidity, driving energy flow and nutrient cycling in these ecosystems [30]. Members of this class are characterized by their ability to oxidize ferrous iron and/or reduced inorganic sulfur compounds (RISCs) as electron donors, and to use oxygen or ferric iron as electron acceptors under oxic or microaerophilic conditions [31]. All species exhibit strict chemolithoautotrophy and most thrive in highly acidic environments. Additional electron donors for the class include molecular hydrogen and hydrogen sulfide, underscoring their metabolic versatility [32, 33]. Recent studies have established the taxonomic and functional breadth of *Acidithiobacillia* [34, 35], yet different aspects of their ecology are less well understood. These include the roles of dispersal and colonization in shaping *Acidithiobacillia* persistence in acidic environments and the effect of fine-scale microhabitat partitioning (e.g. free-living vs. particle- associated communities) and temporal dynamics (e.g. diel cycles, seasonal variations) on microbiome structure. Both aspects are particularly important in mountain rivers such as RAS, with nivo-pluvial hydrological regimes, characterized by relevant seasonal and diel variations in water flow and transported sediment (e.g. [36, 37]). Such variations can differentially impact free-living and particle-associated communities by modulating dispersal dynamics, nutrient availability, and the residence time of particles, potentially shaping distinct microhabitat-specific successional trajectories. While recent evidence indicates that suspended particles in aquatic ecosystems act as hotspots for microbial interactions and may host functionally distinct assemblages compared to the surrounding water [38–40], studies specifically targeting suspended particles in acidic volcanic rivers (and elemental sulfur particles in particular) remain scarce. This is particularly relevant in the context of mineral interacting microbial systems such as the one in RAS [41]. Understanding these eco-evolutionary dynamics is essential to uncover the mechanisms by which *Acidithiobacillia* influence community assembly in acidic ecosystems, thereby informing targeted strategies for microbial resource management and biotechnological applications in extreme environments. Here, we examine community assembly across water-column microhabitats (free-living vs particle-associated) and sulfur-attached biofilms, using *Acidithiobacillia* as a focal lineage to interpret succession and genomic diversification during substrate colonization under extreme acidic conditions and to understand the ecological processes acting upon *Acidithiobacillia*-dominated microbiomes. We hypothesized that particle-associated communities suspended in the water column of RAS act as reservoirs of microbial diversity due to the stabilizing effect of surface attachment, and therefore exhibit reduced short-term variability relative to free-living communities across diel sampling. We further predicted that free-living communities would exhibit stronger diel variation across flow conditions, in response to shifts in mixing, transport, and source contributions within the water column, rather than rapid *in situ* population turnover. To test these hypotheses, we used a dual experimental approach that integrates *in situ* and *ex situ* assays to disentangle the roles of environmental filtering, dispersal, and historical contingency in shaping community assembly. We characterized community structure across free-living and particle-associated fractions under contrasting flow regimes and across the diel cycle. In situ experiments captured natural colonization dynamics under real-world hydrological and chemical fluctuations, while the *ex situ* laboratory recolonization (microcosm) assays examined how colonization history and biotic interactions shape succession under controlled conditions. Together, these complementary approaches allowed us to distinguish the relative contributions of deterministic (e.g. selection, priority effects) and stochastic (e.g. dispersal, drift) processes structuring microbial communities in acidic rivers, while also enabling higher-resolution interpretation of *Acidithiobacillia* population dynamics within the broader community context.

## Materials and Methods

### Sample collection and field procedures

Sampling was conducted at Cascada de la Culebra (CC), an acidic waterfall located in the Rio Agrio Superior (RAS) at 1690 m.a.s.l., within the Copahue-Caviahue Provincial Park early in March 2023. Water samples were collected from high- and low-flow areas of CC, at 8 h, 15 h and 20 h, over 4-days as in [24] (Table S1). Water was pre-filtered through 8 μm Whatman grade 2 cellulose filters discs (assigned as particle-associated community or attached fraction, AF) and collected on 0.22 μm Sterivex filters (Millipore) (assigned as free-living community or planktonic fraction, PF) for amplicon sequencing (2 L). For bulk water sample (25 L) metagenomic sequencing water was filtered through 0.22 μm MCE membrane disc filters (Millipore) using a 500 ml Nalgene serial vacuum filtration system. Filters were stored at −20°C in the field and thereafter at −80°C until DNA extraction and sequencing. Physicochemical parameters including pH, temperature, electroconductivity, and others (Table S2) were recorded *in situ* at three time points over a 4-years period (February 2019, February 2020, March 2023), using a portable multiparameter meter (Hanna HI 9829). Elemental composition of filtrated water samples (1 L) was determined by atomic absorption following acid digestion at UNComahue [42]. Sampling zones were delineated by their flow-induced physical disturbance: high-flow areas at RAS-CC were identified by visible surface turbulence, eddy formation, and splashing at the plunge-pool outlet; low-flow zones exhibited smooth, laminar-like water with minimal surface disruption, reduced sediment suspension, and slower particle movement (Supplementary Videos S1–S2). Sediments and rock bed were intentionally excluded based on prior evidence of their low prokaryotic abundance and algal-dominated composition, in contrast to the acidophile-rich water column [43, 44]. Water flow was measured using a Global Water FP111 flow probe (accuracy ± 0.1 m/s). Measurements were taken at each sampling location at a depth of ~10 cm below the surface. Three replicate readings were recorded along the central current line 4at each site, and mean velocities were calculated.

### Study design overview

To investigate microbial community assembly processes in an extreme acidic river system, we employed a two-pronged experimental strategy combining *in situ* colonization and *ex situ* recolonization assays using sulfur beads (S-beads) as model mineral substrates. The *in situ* colonization experiment allowed us to capture the natural dynamics of microbial attachment and succession under real-world physicochemical and hydrological fluctuations (e.g. diel cycles, sediment transport). The *ex situ* recolonization assay, conducted under controlled laboratory conditions, enabled us to test the effects of historical colonization (priority effects) and biotic interactions on community development across successive colonization cycles. Together, these complementary approaches allowed us to dissect the relative contributions of environmental filtering (deterministic processes), dispersal, and historical contingency (stochastic and priority effects) in structuring particle-associated microbial communities.

In situ colonization experiment of S-beads were performed in March 2023 (Table S1). Sulfur beads (S-beads) were prepared as in [45]. Flow-through columns containing equal amounts of sterile quartz (as inert support) and sulfur (20 g each/column) of similar granulometry (3–4 mm) were placed inside a custom holder adapted from a polypropylene test-tube rack designed for Falcon-size tubes, and submerged 15 cm below the air–water interface, aligned with the water flow, in a low-flow section of the CC plunge pool for 4 days (Fig. S1). This packaging ensured consistent exposure across substrates and liquid flow through the columns [46]. Columns were retrieved at 24 h, 48 h, 72 h, or 96 h, S-beads were recovered under sterile conditions, and washed with acidified water (pH 2.5) before storage at 4°C. Upon collection, S-beads were manually separated from quartz particles and transferred into sterile Falcon tubes containing site-filtered water. Samples were stored at 4°C for up to one week with tube caps loosely screwed to maintain oxic conditions. Only S-beads were processed for subsequent cell detachment and molecular analyses. Cells were detached from the S-beads (10 g) by gentle agitation through repeated inversion in the presence of 0.05% Triton X-100, counted using an Improved Neubauer chamber (Marienfeld GmbH & Co) under an optical microscope (Olympus CX31) with phase contrast, and filtered through 0.22 μm MCE membrane disc filters (Millipore). Filters were stored at −80°C for subsequent DNA extraction. Water pH and temperature were monitored throughout the course of the experiment (Fig. S1B; Table S2).

*Ex situ* recolonization assays of S-beads were specifically designed to extend the *in situ* observations by testing how pre-colonized microbial communities (from 24 h and 48 h *in situ* time points) initiate and influence subsequent colonization trajectories. By using both attached (AF) and planktonic (PF) fractions as inocula in replicated laboratory conditions, we aimed to quantify the influence of colonization history and interspecific interactions, key drivers of priority effects and deterministic assembly, across successive colonization cycles. Experiments (Fig. S2) were conducted under controlled laboratory conditions using minimal saline medium with trace elements (MSM + TE, pH 2.5 [47]). Sulfur beads (S-beads) retrieved after 24 h, 48 h, and 72 h of *in situ* colonization were transported at 4°C and stored under refrigeration for up to one week prior to the *ex situ* laboratory recolonization assays, using either the attached (AF) or planktonic (PF) microbial fractions. These fractions served as inoculants for subsequent recolonization cycles. In situ colonized S-beads were mixed 1:1 with sterile S-beads to evaluate microbial transfer and subsequent colonization. Alternatively, sterile S-beads (5% w/v) were exposed to the planktonic fraction and fresh MSM + TE (1,1 v/v). Recolonization was carried out over 3 consecutive cycles, each lasting 3 days, under batch culture conditions (500 ml Erlenmeyer flasks containing 100 ml of medium), and stable incubation temperature (30°C, 80 rpm). This temperature was selected to match the thermal growth range of most frequent taxa at RAS-CC and to promote efficient recolonization within a practical timeframe. All incubation and sampling were performed under oxic conditions through gentle agitation; organic carbon was deliberately excluded from the recolonization medium to better emulate the oligotrophic and conditions physicochemical of the native system. Cells were harvested for DNA extraction as above. Recolonization samples AF-24, AF-48, PF24, and PF-48 were processed for 16S rRNA gene amplicon sequencing and samples AF-72 and PF72 for shotgun metagenomic sequencing.

### DNA isolation, library construction, and sequencing

Cells retained on filters were recovered by washing the membranes with sterile buffer, followed by centrifugation at 8000 × g for 10 min. Resulting pellets (~0.5 g wet weight) were stored at −80°C until DNA extraction. Total DNA extraction was performed following [48], with subsequent purification using the Genomic DNA Clean & Concentrator kit (Zymo Research). DNA was resuspended in 100 μl TE buffer (Tris 10 mM, EDTA 1 mM, pH 8) and DNA integrity, purity and concentration were assessed through gel electrophoresis and spectrophotometry using the Quant-iT PicoGreen dsDNA Assay on a Synergy H1 microplate reader equipped with a Take3 MicroVolume Plate (BioTek Instruments, Inc.). DNA was further diluted to concentrations suitable for sequencing (>30 ng/μl). Library preparation for both shotgun metagenomics and amplicon-based 16S rRNA gene sequencing was conducted at CD Genomics (NY, USA, https://www.cd-genomics.com). DNA was sequenced on a HiSeq 2000 System (Illumina), generating 150 bp paired-end reads with a targeted insert size of 500 bp. Amplicon libraries were generated using primers targeting the V4 region of the 16S rRNA gene using primers 515F (5′- GTGYCAGMGCCGCGGTAA-3′ [49]) and 806R (5′- GGACTACNNGGGTATCTAAT-3′ [50]) as described in [24].

### Sequence manipulations and bioinformatics analyses

Raw sequence quality was assessed using FastQC v0.11.5 [51], with adapter removal and trimming performed using Fastp v0.23.1 [52]. De-noising and taxonomic classification of amplicon sequence variants (ASVs) were conducted in QIIME2 v2021.8 using DADA2 (classify-sklearn) and the Greengenes2 16S rRNA database 2022.10 [53–55]. Alpha diversity (Chao1, Shannon, Heip’s evenness and Berger-Parker’s dominance), beta diversity (Bray–Curtis dissimilarity) indexes and rarefaction calculations were computed with the *vegan* v2.6.10 [56] and *EcolUtils* R [57] packages. Statistical differences were assessed via ANOVA followed by Tukey’s HSD post-hoc tests. Community assembly mechanisms were quantitatively assessed using phylogenetic bin-based null model analysis and community turnover metrics implemented in iCAMP [58]. A corresponding phylogenetic tree was constructed from ASV sequences using FastTree v2.1.11 [59]. Deterministic processes were inferred based on the beta net relatedness index (βNRI), derived from phylogenetic diversity. Values of βNRI < −1.96 were interpreted as indicative of homogeneous selection, while βNRI >1.96 denoted heterogeneous selection. Values within the range of |βNRI| ≤ 1.96 were considered stochastic. To further resolve the stochastic component, the Raup–Crick index (RC) based on taxonomic dissimilarity was employed. RC values < −0.95 were interpreted as indicative of homogenizing dispersal, RC > 0.95 as dispersal limitation, and |RC| ≤ 0.95 as ecological drift (undominated processes). The relative contribution of each assembly process across all sample pairs was computed by iCAMP. Statistical differences in process contributions between samples were evaluated using pairwise Wilcoxon tests with Bonferroni correction (*P* < .05).

For shotgun sequencing, reads with a quality score > Q35 were retained, and de novo assembled using SPAdes v3.15.2 built-in in SqueezeMeta v1.5.1 [60, 61]. Downstream analyses including contigs assembly statistics, ORF prediction and annotation, were performed using the SqueezeMeta built-in software and the following databases: GenBank [62], eggNOG [63], KEGG [64], and Pfam [65], updated in October 2023. Predicted ORFs within each metagenome and respective taxonomic annotations were used to calculate number of genes pertaining to each COG functional category, and pairwise functional comparison between samples were performed using 95% confidence intervals, with Benjamini-Hochberg method applied to adjust *P* values (significance level < 0.05). For each metagenome, ORF-level abundances were normalized to reads/transcripts per million (TPM). TPM values of ORFs assigned to the same COG category were summed per metagenome and used to calculate log₂ fold changes between samples for community-wide functional profiles comparisons. Because COG categories provide broad functional groupings, we interpret these analyses as shifts in community-level functional potential, rather than a pathway- or gene-resolved assessment of the specific functions driving the observed enrichment patterns. Binning, bin refinement, and metagenome assembled genomes (MAGs) quality assessment were performed using a custom in-house pipeline designed to generate high-quality, publication-grade genomes. Briefly, contigs were subjected to complementary binning approaches, including composition- and coverage-based methods (MetaBAT2, MaxBin2, and CONCOCT [66–68]), as well as AI-assisted binning tools (SemiBin and Avamb [69, 70]). Resulting bins were refined and consolidated using consensus-based refinement and dereplication strategies implemented in MetaWRAP, DAS Tool, and dRep [71–73]. MAG quality, completeness, contamination, and assembly statistics were evaluated using CheckM, QUAST [74, 75]. Only MAGs meeting standard quality thresholds were retained for downstream analyses [74]. Pairwise Average Nucleotide Identity (ANI) was calculated among all non-dereplicated high- and medium-quality MAGs using PyANI v2.13.1 with the ANIb algorithm [76]. Genomic groups were defined following ANI thresholds from [77]: ≥95.9% for species, ≥99.6% for genomovars, ≥99.9% for strains, and 100% for clonal genomes. To construct the phylogenetic tree, bacterial bac120 and archaeal arc53 marker genes sets for each MAG were identified using GTDB-Tk v2.5.2 [78] with GTDB release R226; and MAGs taxonomy was assigned likewise. Each marker was aligned independently with MAFFT v7.490 [79] using the high-accuracy L-INS-i algorithm, and trimmed with trimAl v1.5.rev0 [80] (−gt 0.5 -keepseqs). Trimmed markers were concatenated into an order-preserving sequence comprising 44 MAGs and 54 marker genes. Maximum-likelihood phylogeny was inferred using IQ-TREE 2.4.0 [81], with ModelFinder identifying GTR + F + I + R4 as the optimal substitution model based on BIC, branch support was evaluated using 10 000 ultrafast bootstrap replicates. Metagenomics sequences used in this study were deposited at the National Center for Biotechnology Information (NCBI) under the BioProject accession ID PRJNA914835.

### Data visualization and manipulation

All analyses were performed with open-source R v4.4.1 through RStudio 2024.04.2 [82]. Summary statistics and figures were computed using R packages and libraries. PCA was conducted using *factomineR* v2.11 and *factoextra* v1.0.7 to evaluate environmental variable contributions [83, 84]. The geographic proximity index (GPI) was calculated using *geosphere* v1.5–20 [85]. Heatmaps, ordination plots, and other visualizations were generated using *ggplot2* v3.5.1, *ggdendro* v0.2.0, *gplots* v3.2.0 and *Rstatix* v0.7.2 [86–89]. NMDS and multivariate analyses were performed using the *factoextra* v1.0.7, *ape* v5.8–1 and *vegan* v2.6–10, *permute* v0.9–7, *minpack.lm* v1.2–4 and *DESeq2* v1.46.0 packages [56, 84, 90–93]. Improvement of vectorial and raster figures was made using *Inkscape* v1.0 (https://inkscape.org) and *Affinity* v2.6.0 (https://affinity.serif.com).

## Results

### Physicochemical and hydrological factors shape microbial habitats at RAS-CC

**Figure 1:**
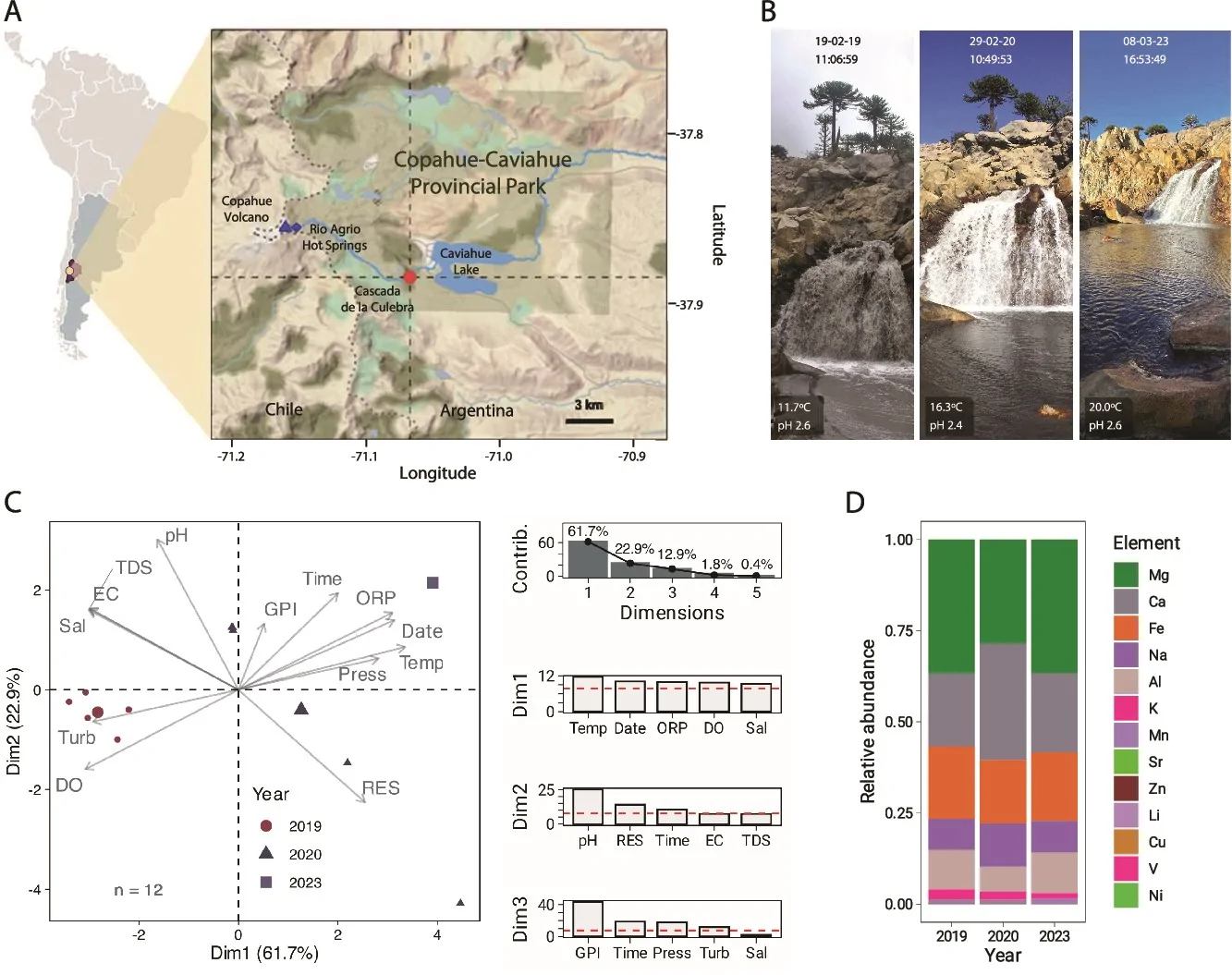
Study area and physicochemical characteristics of the water column at CC. (A) Map of the study area at the Copahue-Caviahue Volcanic Complex located in the Copahue-Caviahue Provincial Park (Neuquén, Argentina), showing the headwaters (blue position marks) and Cascada de la Culebra (red position marks). (B) Representative images of the CC waterfall and plunge pool from three sampling years (2019, 2020 and 2023), including key metadata (average temperature, pH, date and time of sampling). Additional metadata of the samples is provided in Table S1 and Table S2. During sampling water flow velocity varied across the water column and lateral section of the plunge pool at RAS-CC: in the mid-zone (0.3 m depth) it was 0.1 m/s and at the margin (near the riverbed) it was 0.03 m/s. The plunge pool displays spatially distinct microhabitats defined by flow regime (low-flow margins vs high-flow mid-zone) and microbial fractionation (free-living vs particle-attached), as explored throughout the study. (C) Relative abundance of chemical elements in the CC water column. (D) PCA biplot for 12 samples and 13 environmental variables collected at CC, along with the contribution of each variable to data dispersion.

The sampling site, Cascada de la Culebra (RAS-CC), is located at the Copahue-Caviahue Provincial Park within the upper Río Agrio basin (Fig. 1A). RAS-CC is one of four major waterfalls that serve as hydrological regulators, modulating river discharge and sediment transport. The associated plunge pool forms a highly aerated aquatic environment (Fig. 1B; Table S2), with physically distinct microhabitats across the water column (i.e. spatially structured physicochemical niches within the plunge pool, driven by lateral flow gradients and the ecological partitioning between free-living and particle-associated microbial fractions). Across the 2019, 2020, and 2023 sampling campaigns, core physicochemical parameters remained consistent, with acidic pH (2.4–2.6), elevated ion concentrations (average 1465 ppm), and moderate temperatures (11.7–20°C) observed under comparable meteorological conditions (Fig. 1C). No significant differences in total dissolved solids were detected across years (*P* = .79), indicating long-term geochemical stability of the site (Table S2). Principal Component Analysis revealed that interannual variation was driven primarily by temperature and oxidation–reduction potential (Dim1), while diel variability structured Dim2, correlating with pH and conductivity (Fig. 1D). These patterns confirm that despite consistent geochemical baselines, short-term temporal dynamics (i.e. diel cycles) introduce meaningful fluctuations in microhabitat conditions. In parallel, hydrodynamic measurements revealed persistent spatial heterogeneity within the plunge pool: (i) low-flow zones near the pool margins (∼0.03 m/s) were associated with eddy formation and enhanced particle retention; (ii) high-flow central zones (∼0.1 m/s) exhibited laminar and higher-energy currents (Supplementary Videos S1–S2). These results demonstrate that RAS-CC maintains a stable geochemical environment across years but undergoes fine-scale spatial and diel fluctuations in flow and acidity, which are likely to influence microbial community assembly and colonization dynamics across the system.

### α-diversity patterns reveal spatio-temporal partitioning of the RAS-CC microbiome

**Figure 2:**
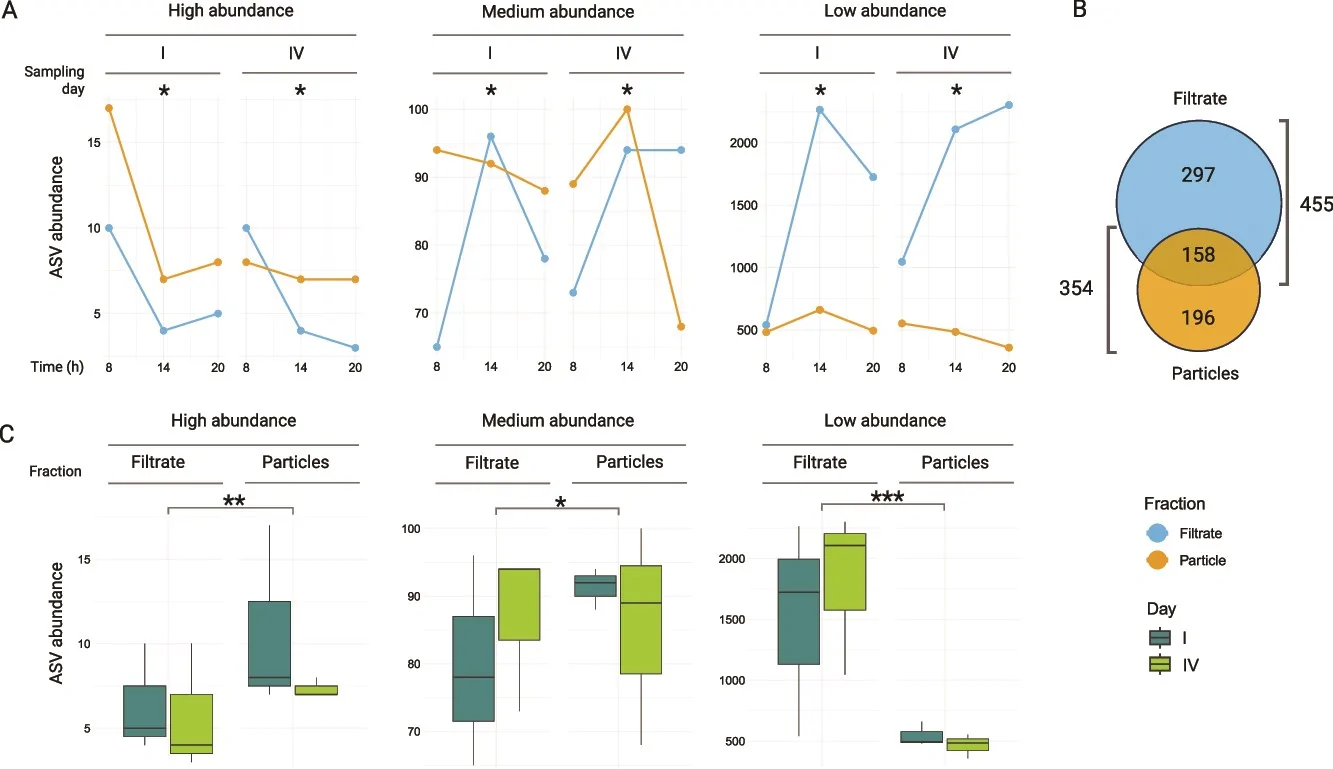
ASV richness comparison between filtrate and particle fractions in the RAS-CC water column. (A) Line plot of observed ASV richness classified by relative abundance categories: high (>1%), medium (≤1% and > 0.1%), and low (≤0.1% and > 0.01%) relative abundance categories, analyzed by fraction (filtrate vs. particulate) and sampling time (8 h, 15 h, 20 h, n = 6 per treatment). ASVs with relative abundance ≤0.01% were considered below detection and excluded from category-based analyses. (B) Shared and fraction specific total ASV abundances in filtrate and particle fractions. (C) Boxplot of the relative abundance per category (high, medium, low), analyzed by fraction (filtrate vs. particulate) across sampling days (I and IV).

We first examined how α-diversity varies across microhabitats and time, focusing on diel fluctuations and spatial partitioning between free-living (filtrate) and particle-associated communities. Alpha diversity metrics based on 16S rRNA gene data are presented in Table S3a. Relevant differences in total ASV counts were observed between fractions (Fig. 2A, Table S3b). Chao1 richness was significantly higher in the filtrate (mean = 1755) than in particles (mean = 769; *P <* .001). Both time of day, and the interaction between fraction and hour, explained additional variance (*P <* .05). Post-hoc pairwise comparisons (Tukey’s HSD test) confirmed consistent higher richness in the filtrate, with peak values observed after noon (*P <* .001). Shannon index values mirrored these trends, being significantly influenced by the fraction and its interactions with time (*P <* .05). In contrast, Heip’s evenness and Berger-Parker’s dominance showed no significant effects from fraction, hour, or their interactions, suggesting that the dominant taxa within each fraction remained relatively stable across the diel cycle. To further resolve these trends, we partitioned ASVs by relative abundance into high (>1%), medium (≤1% and > 0.1%), and low (≤0.1% and >0.01%) categories; ASVs at ≤0.01% relative abundance were considered below detection. After singleton filtering, 3073 ASVs were retained, of which 347 were shared across fractions and timepoints (Fig. 2B). These core ASVs were consistently more abundant than fraction-specific ASVs, indicating partial ecological overlap and suggesting that transient or niche-adapted taxa likely account for fraction-specific composition. High-abundance ASVs were similarly represented across filtrate and particle fractions, while low-abundance ASVs displayed higher relative abundance in the filtrate (Fig. 2C), suggesting a greater contribution of rare and potentially transient taxa to the free-living assemblage. To further explore how hydrodynamic forces influence microbial diversity and structure across free-living and particle-associated fractions, we performed targeted sampling of contrasting flow regimes within the plunge pool.

### Water flow shape α-diversity patterns across microbial fractions at RAS-CC

**Figure 3:**
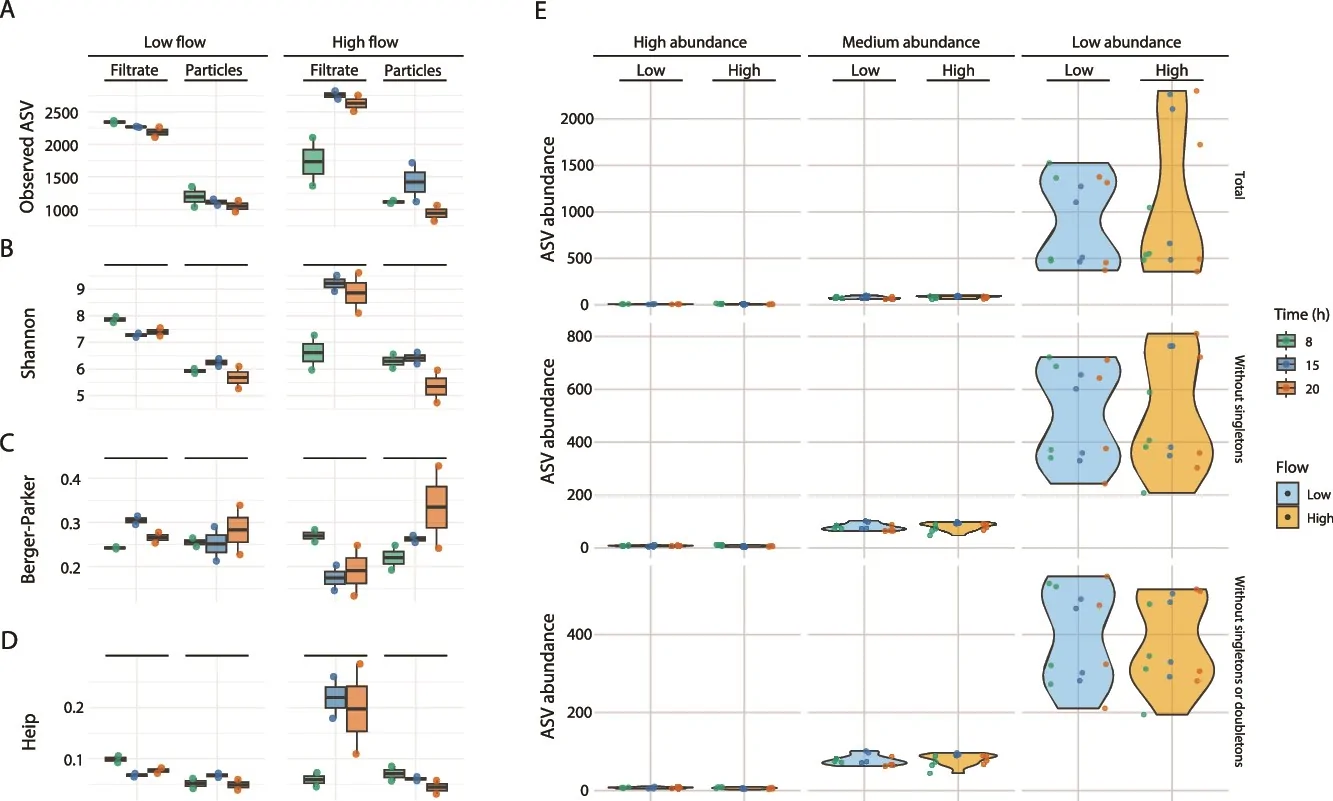
Effects of water flow on microbial richness and diversity metrics at RAS-CC variation in Chao1 richness (A) and Shannon index (B) under different flow conditions. (C) Berger-Parker’s dominance index. (D) Heip’s evenness index. (E) Variability in microbial diversity metrics across high-, medium-, and low-abundance taxa. ASVs were analyzed in filtrate and particle fractions under high-flow (0.2 m/s) and low-flow (0.03 m/s) conditions, evaluated at different diel cycle time points (dawn, midday, dusk). Statistical analysis using ANOVA: (A, B) *P <* .05, (C) *P* > .05, (D) *P <* .05.

To disentangle the influence of water flow on microbial community structure, we compared diversity patterns across two plunge pool zones with distinct flow velocities and evaluated their variation across the diel cycle. Mean flow velocity differed consistently between sites, averaging 0.10 ± 0.01 m s^−1^ at the high-flow zone and 0.03 ± 0.005 m s^−1^ at the low-flow zone. Although diel variation in flow was hypothesized, point velocity measurements collected during the austral summer sampling window, under stable hydrometeorological conditions, did not detect significant within-day fluctuations. Both richness and diversity metrics were therefore evaluated across diel timepoints within these fixed flow regimes (Table S3). Analysis of variance (ANOVA) of the ASVs richness (Chao1) indicated minimal direct effects of flow, but significant flow:hour and fraction:flow:hour interactions (Fig. 3A, *P <* .05). In turn, the Shannon index value was significantly influenced by flow (*P <* .05) and its interaction with time (*P <* .001), with marginal effects from fraction (Fig. 3B). Berger-Parker’s dominance was unaffected by any main factor or interaction (Fig. 3C), while Heip’s evenness was shaped by a three-way interaction (fraction:flow:hour; *P <* .05), with high-flow filtrates showing significantly elevated evenness at noon and dusk (Fig. 3D). Within-group variability in these different diversity metrics was mostly contributed by low abundance taxa, regardless of fraction, flow velocity or diel cycle (Fig. 3E). On this basis, we next assessed whether community composition differed systematically between free-living (filtrate) and particle-associated fractions, and whether these differences were driven primarily by dominant taxa or by shifts across abundance-ranked ASV classes.

### Taxonomic partitioning between free-living and particle-attached fractions at RAS-CC

**Figure 4:**
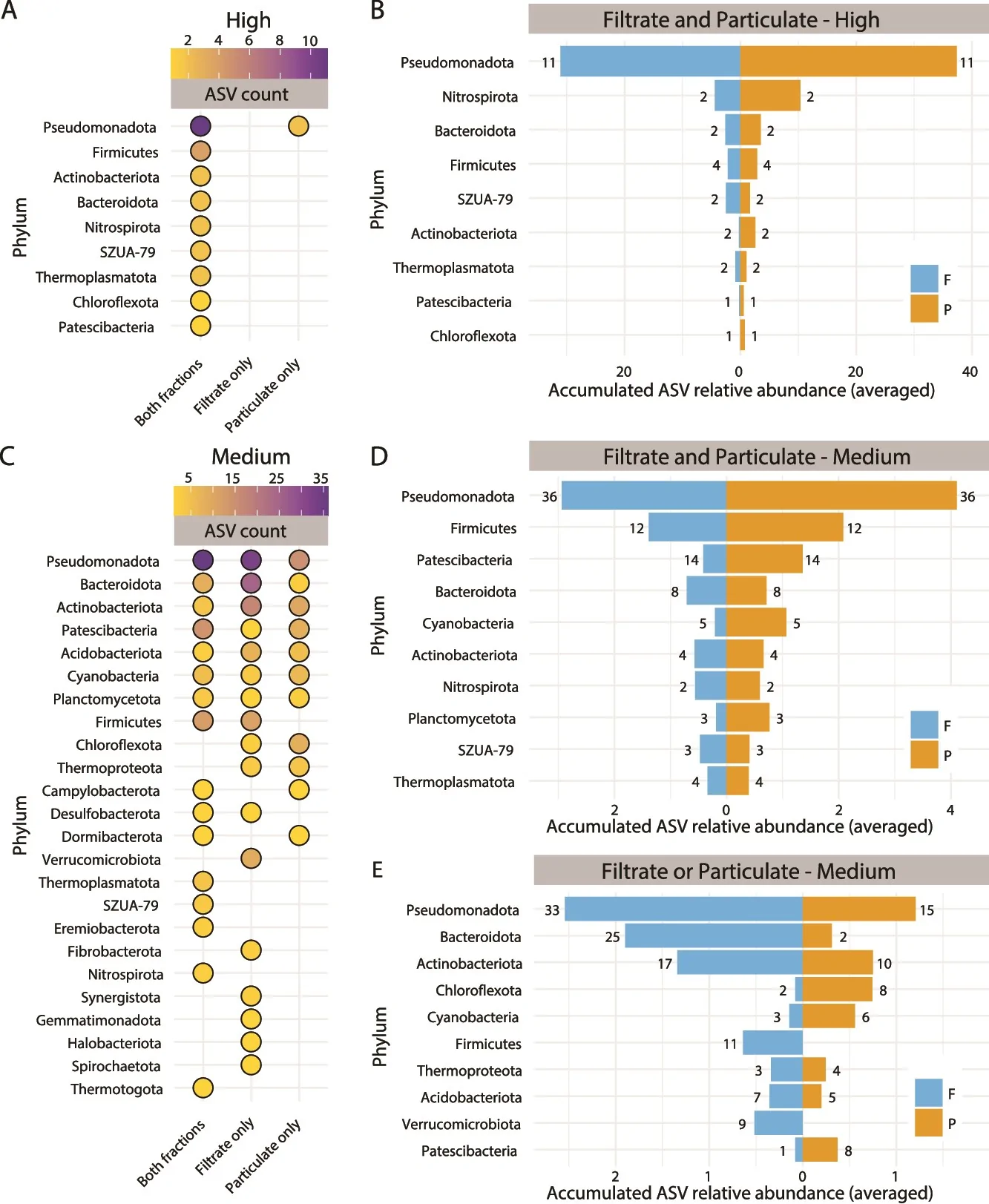
Taxonomic composition and fractional partition of the RAS-CC microbiome. (A, C) dot plot of the count of high- (>1%; A) and medium-abundance (>0.1%; C) ASVs per phylum detected in the filtrate and particle fractions. (B, D) Back-to-back bar chart depicting the cumulative relative abundance of ASVs per phylum in the filtrate and particle fractions, for high (B) and medium (D) abundance taxa. (E) Back-to-back bar chart depicting the fraction-exclusive cumulative relative abundance of ASVs per phylum in either the filtrate or particle fraction for medium abundance taxa. ASVs in the low-abundance range (0.01%–0.1%) are shown separately in Fig. S3.

Microbial composition varied significantly between filtrate and particle-associated fractions. A total of 741 core ASVs (defined as those consistently present in at least 50% of experimental replicates, Table S4) were identified, comprising 715 bacterial and 26 archaeal ASVs spanning 36 phyla. High-abundance ASVs (>1%) were largely shared (70%) between fractions (Fig. 4A), while low-abundance ASVs tended to be fraction-specific (Fig. S3). Among the nine phyla with the highest overall abundance, *Pseudomonadota* members dominated both filtrate and particle-associated fractions across timepoints and flow conditions, largely driven by the prevalence of the a sulfur- and iron-oxidizing/reducing autotrophic species *Acidithiobacillus ferriphilus* [94] (Fig. 4B; Table S4). Other high-abundance ASVs showed fraction-specific patterns (Table S4), with *Leptospirillaceae*, *Acetobacteraceae*, and *Acidimicrobiaceae* being more prevalent in the suspended particles, and *Marinilabiliaceae* and *Comamonadaceae* more abundant in the filtrate. The differential representation of these taxa across fractions, including several with previously described roles in iron and sulfur cycling [95], indicates that microhabitat conditions may shape microbial assemblages structure in ways that are consistent with functional niche partitioning, although direct activity measurements were not assessed in this study. ASVs of intermediate abundance (>0.1%, 294 ASVs, 24 phyla) exhibited broader taxonomic and ecological diversity, including known acidophiles, acid-tolerant, and neutrophilic lineages, along with greater variations fold abundance (Fig. 4C and D, Table S4). Among particle- exclusive taxa (244 ASVs, 26 phyla), the most abundant were ultrasmall Patescibacteria (>30% of the total), alongside diverse members of *Pseudomonadota*, *Bacteroidota*, *Actinobacteriota*, *Chloroflexota*, *Cyanobacteriota*, and various unclassified ASVs (Fig. 4E, Table S4). In contrast, filtrate-specific taxa (336 ASVs) spanned 22 phyla, with *Pseudomonadota*, *Bacteroidota*, *Firmicutes*, *Actinobacteriota*, and *Acidobacteriota* representing >75% of the taxa present in this fraction (both in terms of ASV counts and accumulative abundance; Fig. 4E, Table S4). Partitioning patterns observed for low-abundance taxa (0.01%–0.1%) are shown in Fig. S3. Given the greater stability of microbial communities associated to particles and the observed compositional differences between fractions (Fig. 4, Fig. S3, Table S4), we next examined microbial succession during colonization of a defined solid substrate deployed under natural flow conditions at RAS-CC.

### Microbial community succession during *in situ* sulfur beads colonization at RAS-CC

**Figure 5:**
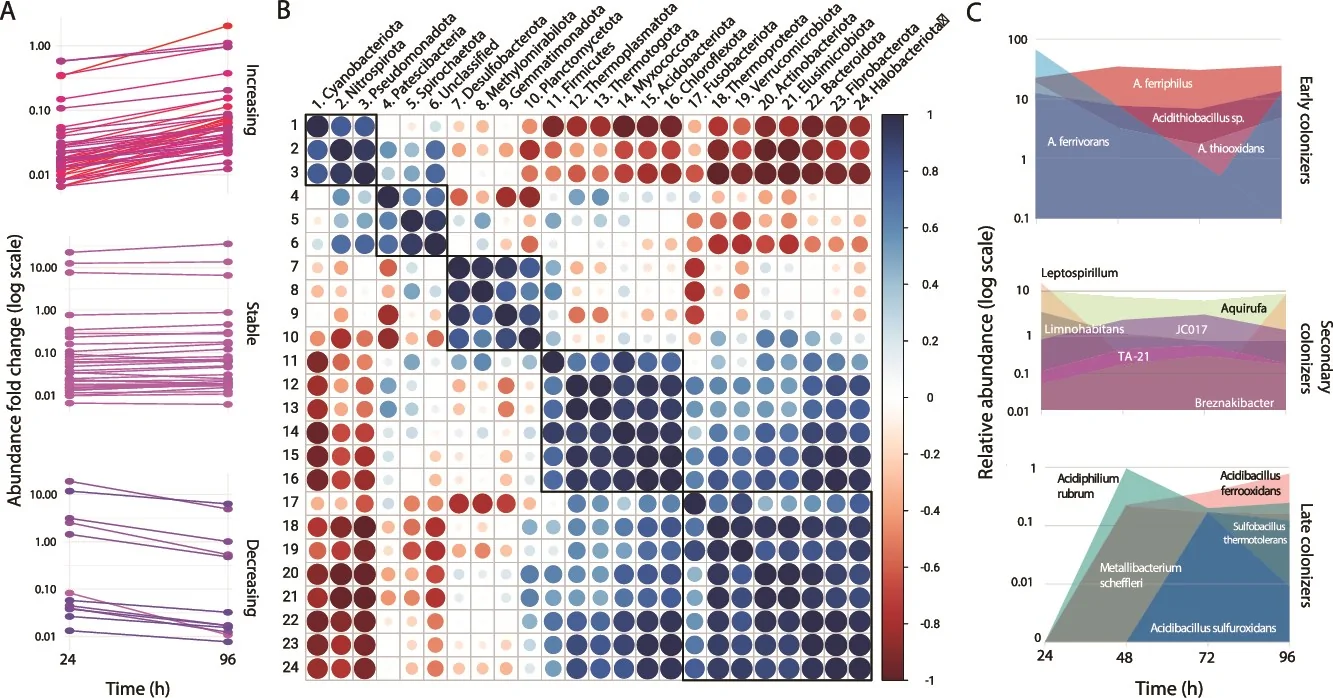
Successional dynamics and ecological roles of key taxa in S-bead colonization at RAS-CC. (A) Relative abundance trajectories of individual ASVs spanning high, medium, and low abundance categories across early (24 h), intermediate (48–72 h), and late (96 h) colonization stages. Each line represents a single ASV, which was classified as increasing, stable, or decreasing based on its temporal trend. The full list of ASVs, including their temporal patterns, abundance classifications, and taxonomic assignments can be found in Table S5. (B) Correlation analysis of phyla-level taxa, showing clusters with positively correlated occurrence and abundance patterns across colonization stages. (C) Representative primary, secondary, and late colonizers identified during the stepwise microbial succession pattern in sulfur-driven community assembly at RAS-CC. Timeline spans 24–96 h.

To assess microbial community assembly on mineral substrates under natural flow conditions, sterile sulfur beads (S-beads) were used as ecologically relevant surrogates for the dominant sulfur-particles in the system. S-beads were deployed *in situ* for 96 h (Fig. S1A). Attached-cell counts increased steadily, peaking at 1.68 × 10^8^ cells/g of sulfur after 72 h, before slightly declining at 96 h (Fig. S1B). The ASV counts mirrored the trends in cell counts, with richness (Chao1) being lowest at 24 h (pioneer colonization) and peaking at 72 h (Table S5). Heip’s evenness also increased from 24 h to 72 h, reflecting greater community balance over time. The concurrent rise in diversity and biomass during the early stages indicates that succession involved both active colonization and population growth. Together, these patterns reveal a dynamic trajectory: an initial colonization stage (24 h), followed by community stabilization (48–72 h), and a later decline in both diversity and biomass by 96 h. Out of 4874 ASVs retained for the analysis, 13 dominant ASVs from 4 bacterial phyla (*Pseudomonadota*, *Bacteroidota*, *Actinobacteriota*, and *Cyanobacteriota*) accounted for 49%–90% of total abundance across timepoints (Table S5). Six of these ASVs increased or remained abundant throughout the experiment, while others showed declining trajectories (Fig. 5A). Similar increasing, stable, or decreasing patterns were also observed among medium- and low-abundance ASVs. Phylum-level co-occurrence patterns provide a higher-order overview of coordinated trajectories across many ASVs and reveal clusters of taxa with positively correlated dynamics during colonization (Fig. 5B), including highly and stably abundant *Pseudomonadota* (e.g. *A. ferriphillus*), *Nitrospirota* (e.g. *Leptospirillum ferrooxidans*) and *Cyanobacteriota*. To further anchor the emerging successional trajectories, Fig. 5C highlights representative ASVs discussed at the lowest reliable taxonomic rank, including species-level assignments where supported. The stronger dominance of a small subset of ASVs in S-beads (Table S7) supports this finer-grained interpretation and facilitates trait-informed ecological inference for key taxa, grounded in well-established species descriptions and experimentally validated physiological traits. **Early colonizers** included sulfur- and iron-cycling autotrophic *Acidithiobacillus* spp. (i.e. *A. ferriphillus* [94] and *A. ferrivorans* [96]), the obligate iron-oxidizer *L. ferrooxidans* [97], and the heterotrophic freshwater bacterium *Aquirufa antheringensis* [98], along with other transient taxa that declined after 48 h (Fig. 5C). Declining taxa included *Polynucleobacter acidiphobus* [99] and *Limnohabitans* sp. [100], along with ASVs affiliated with 13 additional phyla, all with low relative abundance (<0.01%; 49 ASVs). During the stabilization phase (48–72 h), communities featured taxa of medium (>0.1%) or low (>0.01%) abundance (79 ASVs pertaining to 12 phyla, Fig. 5A), including *Acidibacillus ferrooxidans* (now *Ferroacidibacillus* [101]), *Breznakibacter xylanolyticus* [102], along with increasing ASVs assigned to *Acidithiobacillus thiooxidans* [103], *Nitrososphaeraceae* sp. TA-21 [104], and uncultured bacteria from 4 phyla (Table S5). These trajectories are consistent with **secondary colonizers** exploiting niches created by early colonizers under *in situ* conditions (Fig. 5C). In turn, **late colonizers** (detected in the S-beads at 48 h or thereafter) included a highly diversified set of medium (11 ASVs) and low abundance (145 ASVs) taxa (Table S5), supporting specialized ecological roles within the mature biofilm formed during *in situ* colonization of the S-beads. We next performed *ex situ* laboratory recolonization experiments under controlled laboratory conditions to evaluate the persistence and turnover of microbial consortia in the absence of environmental variability.

### Succession dynamics in sequential S-beads *ex situ* recolonization experiments

**Figure 6:**
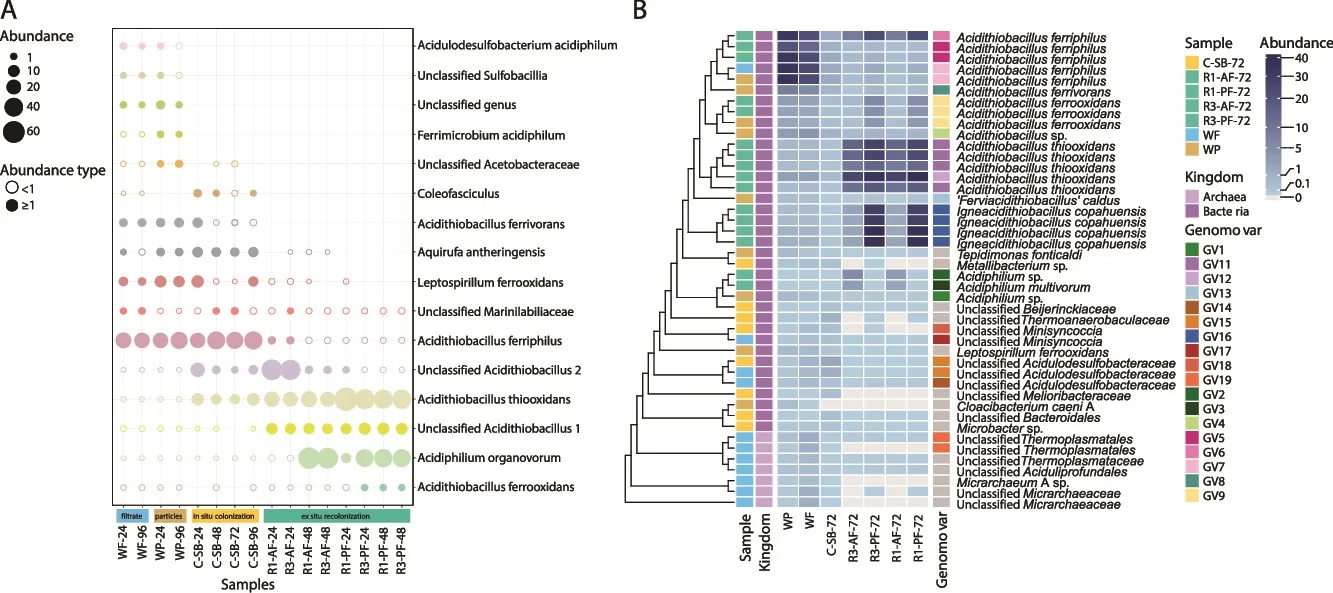
Shifts in microbial community diversity and composition across colonization experiments. (A) Abundance trajectories of high-abundance ASVs (>1%) across experiments: filtrate (light blue) and particle (light brown) samples (averaged per day), in situ colonized S-beads (yellow) and *ex situ* recolonization experiments (green). ASVs are classified to the highest taxonomic rank available and ordered according to their abundance trajectory in the sample to highlight abundance shifts (>1%, filled circles; <0.1% void circles), and replacements. Datapoints of relative abundance below 0.01 were not considered in this analysis (n.d. = no detected). (B) Genome-resolved patterns of microbial succession during S-bead recolonization with relative abundances across samples displayed as a heatmap. MAGs were clustered according to a maximum-likelihood phylogenomic tree and grouped into genomovars using an ANI threshold of ≥99.6% (≥99.9% for strain-level variants) to resolve fine-scale microdiversity within dominant taxa.

To evaluate how colonization history influences microbial succession under controlled conditions, we conducted three sequential recolonization cycles *ex situ* (R1, R3) using sterile S-beads inoculated with attached (R0_AF) or planktonic (R0_PF) fractions of S-beads colonized *in situ* for 24 h or 48 h (R0) (Fig. S2; Table S6). While total ASV richness (Chao 1) was largely unaffected by the transition from an open *in situ* to a closed *ex situ* system (Table S3c), Shannon index and Heip’s evenness metrics decreased significantly in the *ex situ* samples (*P <* .01), regardless of the initial inoculum (R0_AF vs. R0_PF, *P <* .05) or the cycle (R1 vs. R3, *P <* .05; Table S3d; Table S7). This was accompanied by increased dominance of *Acidithiobacillaceae* and *Acetobacteraceae*, particularly in R0_PF-derived communities (Fig. S4). To further assess between-treatment community differences, we also evaluated beta diversity using Bray–Curtis dissimilarity. *Ex situ* recolonized communities grouped more closely with *in situ* S-beads than with water fractions, indicating ecological similarity shaped by substrate and mode of colonization (Fig. S4; Fig. S5). This pattern was supported by PERMANOVA (Table S8), which identified significant compositional effects of fraction (attached vs planktonic; *R*^2^ = 0.29, *P =* .001) and inoculum stage (24 h vs 48 h; *R*^2^ = 0.17, *P =* .007). Transfer history accounted for the largest compositional shift, with R0 differing strongly from both R1 and R3 (*R*^2^ = 0.55–0.57, *P =* .001), whereas R1 and R3 were not detectably different (*R*^2^ = 0.03, *P =* .53), consistent with rapid restructuring after transfer followed by compositional stabilization across subsequent cycles. Difference in the number of high- and medium-abundance ASVs between treatments were evident (Fig. 6A; Table S6). ASV-level shifts included the rise of *A. thiooxidans* (~23% in attached communities and ~50% in planktonic communities) and *Acidithiobacillus* spp., and the decline of *A. ferriphilus*, the dominant *in situ* taxon. Succession also differed with the species pool: 24 h inocula favored early *Acidithiobacillaceae* dominance (>70%), while 48 h inocula led to dominance (>50%) by the obligately heterotrophic acidophile *Acidiphilium organovorum* [105]. Collectively, these patterns are consistent with early, inoculum- and fraction-dependent divergence followed by stabilization across transfers, suggesting a role for historical contingency (priority effects) during *ex situ* recolonization. Genome-resolved metagenomic analysis validated these trends across treatments and cycles at finer resolution. Of a total of 44 high-to-medium quality MAGs recovered (Table S9), 20 belonged to *Acidithiobacillia*, matching ASV-level dominance patterns and confirming this class as the core colonizers across conditions. Within *Acidithiobacillia*, *A. ferriphilus* persisted throughout the experiment despite declining relative abundance and was represented by three distinct genomovars (ANI ≥ 99.6% [77]). These genomovar exhibited microhabitat-specific distributions, suggesting ecotypic partitioning (Fig. 6B). In contrast, *A. ferrooxidans* was detected at low abundance and represented by a single genomovar, indicating uniform ecological behavior. “*Igneacidithiobacillus copahuensis*” emerged to detectable levels only during recolonization, represented by nearly identical strains (ANI ≥ 99.9% [77]), while *A. thiooxidans* displayed two genomovars segregated across attached and planktonic fractions. Having established the ASV- and MAG-based patterns across treatments and cycles, we next evaluated community assembly processes using the null-model framework.

### Ecological drivers of microbial community assembly across RAS-CC microhabitats

**Figure 7:**
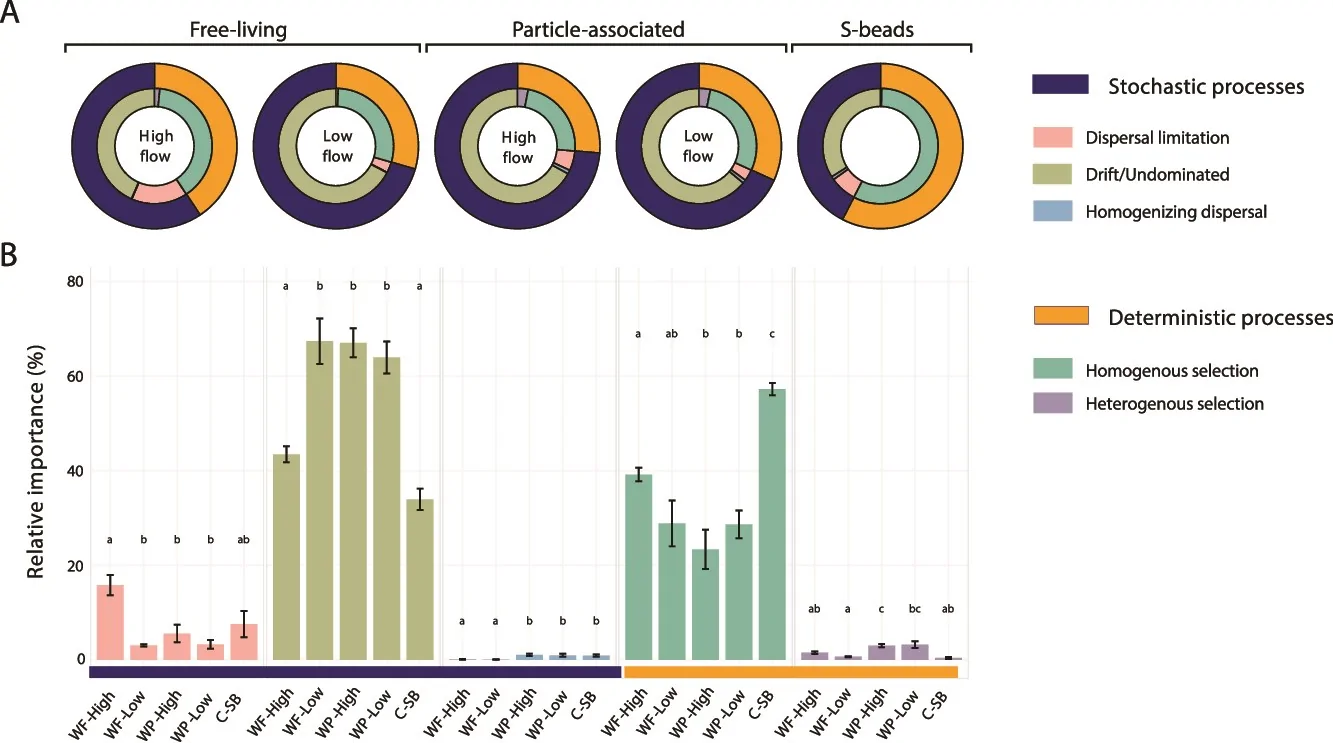
Ecological processes driving community assembly across microhabitats. (A) Relative contribution of deterministic and stochastic ecological process to community assembly within different microhabitats at RAS-CC (n = 4, per sample; averaged valued for replicates). (B) Differences in the relative importance of each process across microhabitats. Different letters indicate statistically significant differences between groups, as determined by pairwise Wilcoxon tests with Bonferroni correction (*P <* .05) with *ab* and *bc* indicating non- significant differences and distinct letters (*a*, *b*, *c*) showcasing statistically significant differences.

Quantification of assembly processes based on community turnover using phylogenetic bin-based null model analysis (iCAMP) revealed significant differentiation among microhabitats (*P <* .01), with distinct mechanistic patterns emerging between free-living, particle-associated, and S-beads attached microbial communities (Fig. 7; Table S10). This variation in assembly mechanisms suggests that environmental heterogeneity at solid–liquid interfaces (operating at the micron-to-millimeter scale defined by suspended particles and S-bead surfaces) can strongly influence microbial community organization within the same plunge pool. Free-living (filtrate) microbiomes showed flow-dependent assembly mechanisms (Fig. 7A), with high-flow communities structured by a balanced combination of drift (43.5%) and homogeneous selection (39.2%), whereas low-flow communities were more strongly dominated by drift (67.4%, *P <* .01). High-flow conditions were associated with higher inferred dispersal limitation (15.8%) than low-flow conditions (3.1%, *P <* .05), consistent with increased flow-driven disturbance (mixing) and shorter residence times in the water column, with community turnover remaining phylogenetically conserved. Particle-associated communities exhibited a strong effect of stochastic processes across flow conditions (Fig. 7B), primarily drift (64.0%–67.1%), with moderate contributions from homogeneous selection (23.4%–28.6%). In contrast, deterministic processes played a major role in structuring S-bead associated communities (Fig. 7C), with homogeneous selection accounting for the largest fraction of inferred assembly (57.2%), followed by drift (34.0%). These patterns were significantly different from those observed in other microhabitats (*P <* .01). These results provide a process-level interpretation of the observed enrichment, indicating repeatable environmental filtering rather than purely stochastic turnover, consistent with substrate-driven enrichment on sulfur surfaces.

### Metabolic signatures of community assembly and substrate filtering

**Figure 8:**
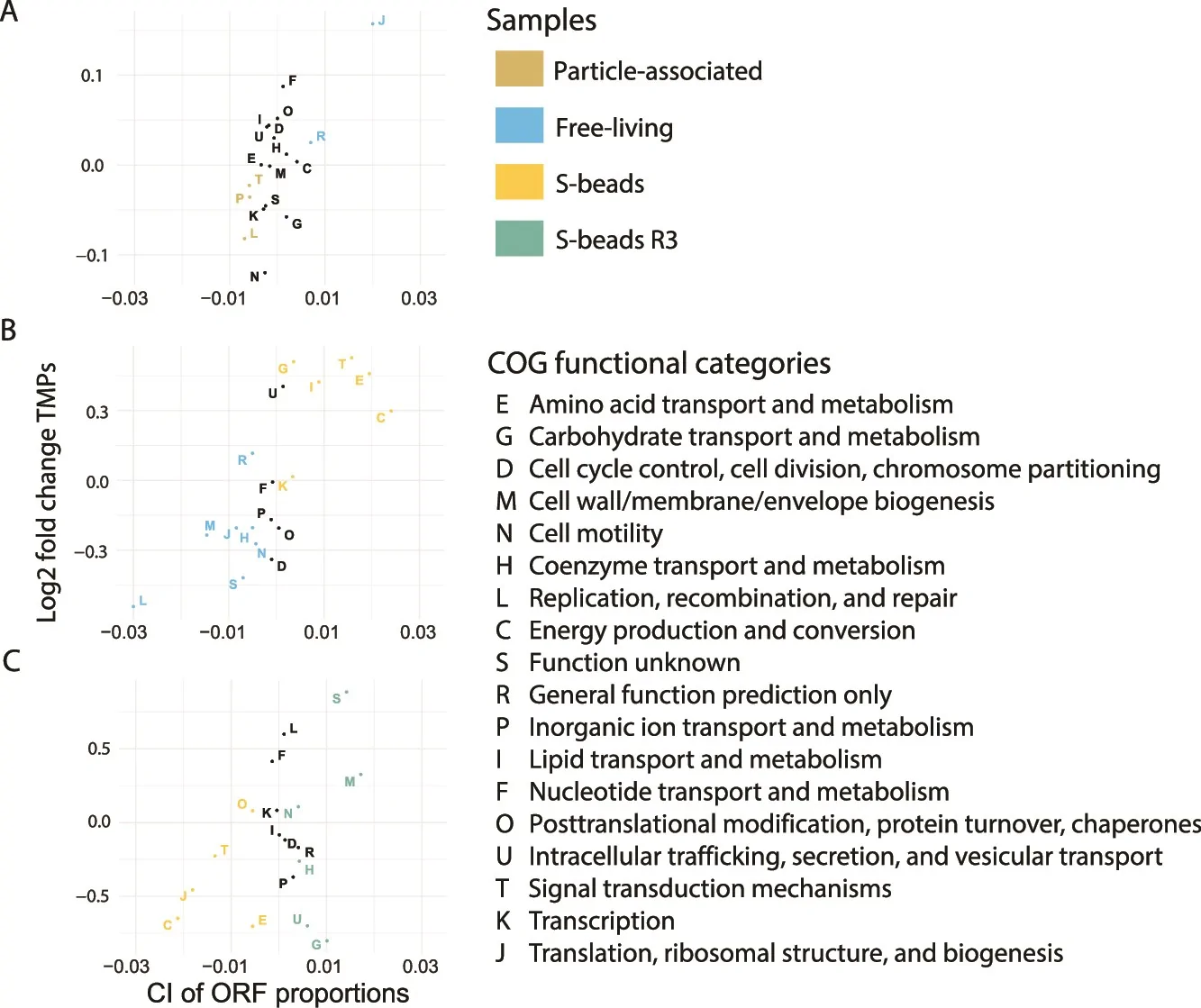
Relative shifts in functional capacity across samples. Scatter plot showing log2 fold changes in total TMP values for COG categories (y-axis), plotted against the confidence interval (CI) of ORF proportions for pairwise comparison of four metagenomes; particle attached, free-living, S-beads and S-beads (R3). Each point represents a COG category and is color-coded by the sample in which that category is significantly overrepresented based on CI analysis (Fig. S6). Black dots denote COG categories with no significant enrichment in any sample. Positive and negative log₂ fold changes indicate relative shifts in representation, aligning with the color assigned by the CI.

We assessed whether functionally distinct traits emerged across microhabitats reflected the underlying ecological assembly processes inferred. To this end we analyzed the broader metabolic landscape captured by metagenome-derived ORF profiles, quantifying COG categories across free-living, particle-associated, *in situ* S-beads, and *ex situ* recolonized S-beads. This ORF-based approach, which circumvented the genome-centric bias of MAGs dominated by *Acidithiobacillia*, revealed clear differences in the composition of the community-wide functional capacity across microhabitats (Fig. 8, Table S9). Free-living fractions displayed a higher representation of genes related to protein-synthesis (J) relative to native particle-associated communities (Fig. 8A, Fig. S6), and in replication, recombination and repair (L), cell wall/membrane/envelope biogenesis (M), cell motility (N), translation (J), and coenzyme transport and metabolism (H) relative to *in situ* S-beads (Fig. 8B, Fig. S6), suggesting a functional profile biased toward cellular proliferation, structural turnover, and dispersal-related traits in the planktonic state. Native particle-associated communities exhibited increased representation of ion transport (P) and signal transduction (T) while *in situ* S-beads harbored genes for energy production (C), carbohydrate metabolism (G), and environmental sensing (T), consistent with enhanced capacity for substrate-associated resource acquisition. Together, these differences reflect a shift in functional potential from growth- and mobility-associated capacities in free-living cells toward sulfur-associated metabolic specialization on S-beads. Recolonized S-beads (R3) showed reduced representation of COG categories associated with central metabolism and information processing (J, C, E, T) relative to *in situ* S-beads (Fig. 8C, Fig. S6), consistent with a more restricted functional repertoire in the recolonized assemblages. In contrast, R3 communities were enriched in cell wall/membrane/envelope biogenesis (M) and unknown functions (S), consistent with selective environmental filtering and functional bottlenecks during recolonization. This shift reflects a narrower, surface-adapted functional profile likely shaped by priority effects and substrate-imposed constraints. Notably, COG categories G and U (intracellular trafficking, secretion, and vesicular transport) were overrepresented in gene counts but underrepresented in TPM-normalized abundance, implying that fewer dominant taxa allocate a larger portion of their genomes to these functions supporting selective retention of traits relevant for substrate utilization and cell–surface/biofilm-associated lifestyles on sulfur beads.

## Discussion

In this study, we examined microbial partitioning, colonization dynamics, and community succession in an acidic riverine system of northern Patagonia, volcanic in origin, that features extreme characteristics (pH < 3). This was done by integrating spatial fractionation analyses with *in situ* and *ex situ* community assembly experiments, and taking into account both hydrochemical and hydrological characteristics of the river at one representative point along its course, RAS-CC. This site featured physicochemical stability over a four-year period providing a consistent environmental framework for the study of community assembly processes. The results revealed that water flow, particle association, and sulfur surfaces drive distinct microbial community assembly patterns with varying degrees of taxonomic and ecologic overlap. Hydrodynamic conditions and spatial fractionation played significant roles in shaping microbial diversity and richness, while temperature, redox potential, and diel fluctuations emerged as key drivers of microbial distribution.

Measured water-column velocities (~0.03–0.10 m s^−1^) provided a coarse proxy for hydrodynamic conditions at RAS-CC, and together with diel variability, emerged as a key physical driver shaping short-term microbial community dynamics across the plunge-pool. Here, free-living microbial communities showed sensitivity to diel changes and flow regime, a pattern more consistent with shifts in the relative contribution of microbial source pools (e.g. upstream inputs, margins, particle-associated reservoirs), mixing intensity, and transport pathways than with rapid *in situ* population turnover. In contrast, particle-associated communities showed lower richness, reduced temporal variability, and a distinct taxonomic composition, consistent with selection for particle-attached lifestyles and particle-associated resource use, rather than short-term hydrodynamic variability alone. These observations align with ecological expectations for riverine systems, in which disturbance regimes and hydrological connectivity regulate community composition through a combination of dispersal, resuspension, and selective retention affecting both stochastic dispersal and deterministic processes [106–109]. At lower flows, increased surface transient storage and local retention of suspended particles may favor short-term persistence of particle-attached assemblages at the particle-scale, even if broader plunge-pool hydraulics remain heterogeneous [110, 111]. Conversely, faster, non-laminar flow with turbulence and shear variability can strongly affect microbial ecology by enhancing particle mobility and reducing residence time in the water column, increasing physical connectivity between distinct microbial reservoirs, including benthic surfaces, marginal biofilms, or upstream inputs, and altering attachment–detachment dynamics on particles and biofilms [112]. Under such conditions, episodic resuspension or sloughing of attached biomass can generate apparent drift-like turnover at the temporal resolution of sequencing surveys [113]. Turbulence and shear can also alter encounter rates with surfaces and boundary-layer transport, thereby modulating both the likelihood of establishment (affecting dispersal-related signals) and the strength of environmental filtering on attached communities [114]. Accordingly, we interpret the observed assembly patterns primarily in a comparative sense across microhabitats and flow regimes, and we acknowledge that unresolved fine-scale hydrodynamic heterogeneity introduces uncertainty in separating dispersal, drift-like turnover, and selection in this setting. Future work combining higher-resolution hydraulic measurements (e.g. velocity fluctuations, shear stress) with particle tracking would further constrain these mechanisms.

In line with this fraction-dependent response to hydrodynamics, our fractionation results further indicated that microhabitat conditions consistently structure taxonomic distributions across abundance categories and promote the recurrent enrichment of lineages previously linked to sulfur/iron cycling or organic-matter turnover in particle-associated fractions. These results align with findings at the acidic river Rio Tinto, in Southwest Spain, where sediment-attached communities exhibit distinct and stable compositions enriched in sulfur- and iron-oxidizing taxa, supporting coupled redox transformations within both biogeochemical cycles [10, 115]. Current knowledge of suspended particle composition at RAS is limited, yet existing evidence suggests marked heterogeneity, including high sulfur loads [29], abundant iron-associated minerals [116–118], and minimal organic contributions [119]. The coexistence of chemically distinct sulfur and iron mineral phases implies that particles vary in composition and reactivity, potentially supporting diverse microbial niches. Recent studies have highlighted mineralogical diversity as a key driver of microbial community structure in AMD systems [11, 120], reinforcing the idea that RAS-CC’s heterogeneous particulate matter may create a mosaic of microhabitats shaping microbial community assembly. This mineralogical heterogeneity *in situ* could partly explain the observed differences in successional trajectories between the field-based and controlled experiments, where sulfur beads represent a chemically uniform substrate and thus select for a narrower subset of microbial colonizers.

**Figure 9:**
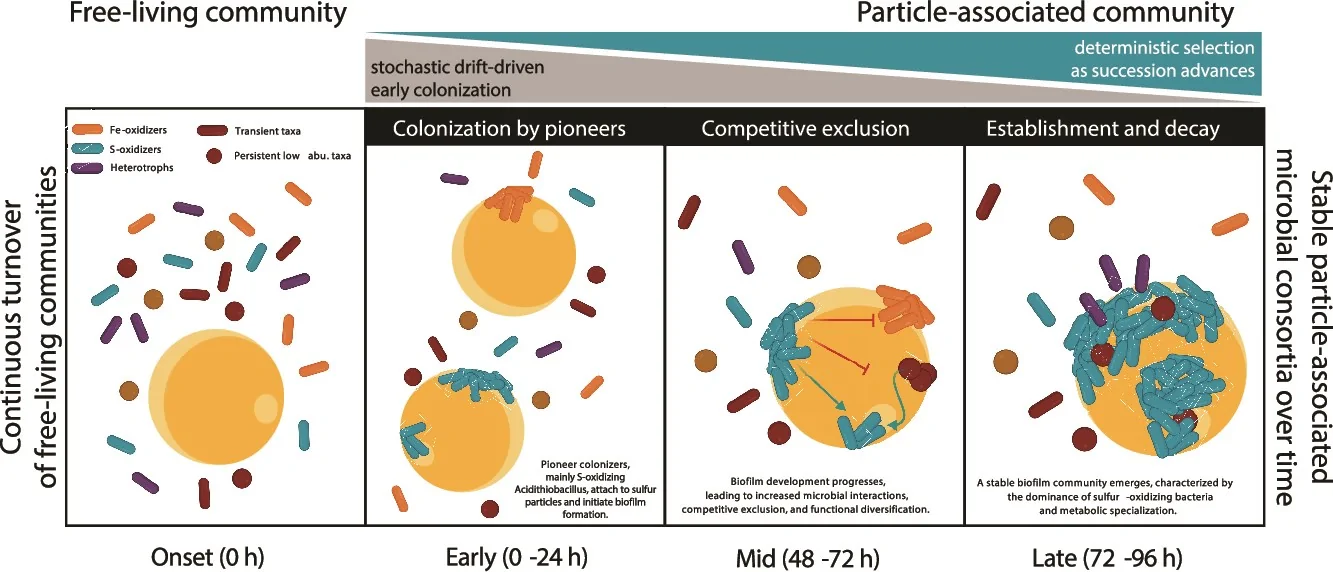
Conceptual model of the microbial succession in sulfur-driven community assembly. S-bead colonization followed a structured trajectory, with distinct early, mid, and late successional stages. Panel 1: Free-living microbial communities including diverse functional groups. Sulfur oxidizers (green), iron oxidizers (orange), heterotrophs (purple), and transient taxa and persistent low-abundance taxa (red). Panel 2: ASV richness increases during early colonization, peaking at 72 h, before declining due to competitive exclusion and environmental selection (Panel 3). Early colonizers (*Acidithiobacillus* spp.) drive sulfur and iron oxidation, stabilizing the biofilm for secondary colonizers (*Acidiphilium rubrum*, *Sulfobacillus thermotolerans*), which exploit metabolic byproducts. Late successional taxa (*Acidibacillus ferrooxidans, Metallibacterium scheffleri*) contribute to biofilm persistence and nutrient cycling. Low-abundance taxa (*Nitrososphaeraceae*) remain throughout, maintaining functional redundancy and ecosystem stability. Ecological interactions are illustrated: red arrows show competitive exclusion, and green arrows represent metabolic interactions (e.g. byproduct utilization). Particle-attached microbes, including biofilm-forming and sulfur-metabolizing taxa, establish stable microbial consortia over time, whereas free-living communities experience continuous turnover. Relative contributions of stochastic and deterministic processes are depicted on top, and the microbial succession timeline is depicted below. Created in BioRender.

In situ colonization of S-beads followed a structured successional trajectory, with microbial biomass and diversity peaking at 72 h before declining at 96 h, mirroring pure culture colonization of S-coupons [121]. This experiment revealed clear successional stages, from early pioneers and transient colonizers to stable and late-establishing taxa with distinct abundance trajectories. Although direct functional activity was not measured, several ASVs could be assigned with high confidence to taxa with well-characterized metabolisms in acidic environments, enabling cautious trait-based contextualization of the temporal patterns. An initial phase of active colonization and biofilm formation was followed by one of competitive exclusion of less-adapted taxa or dispersal of transient taxa, with sulfur-oxidizing bacteria playing a foundational role for later colonizers, mostly heterotrophs (Fig. 9). Medium-abundance secondary colonizers exploited niches created by early pioneers, while specialized late colonizers more transient in nature, likely capitalized on additional microhabitats engineered through the experiment. Overall, the temporal turnover points to shifts in ecological strategy and resource use, consistent with niche modification during biofilm maturation on sulfur particles.

*Ex situ* recolonization assays highlighted the role of priority effects and competitive interactions during S-bead community assembly. Communities seeded with 24 h native sulfur biofilms were dominated by *Acidithiobacillaceae*, which accounted for over 70% of the taxa present. In contrast, those seeded with 48 h biofilms showed a decline in *Acidithiobacillaceae* abundance (~30%) in favor of *Acidiphilium*, which became the dominant taxon (>50%). Although most *Acidiphilium* spp. are obligate organoheterotrophs, some strains have been shown to oxidize reduced sulfur compounds [122], which may provide an energetic advantage under sulfur-rich conditions. Combined with access to organic carbon produced during early colonization, this may explain the competitive dominance of *A. organovorum* observed in S-beads seeded with later-stage communities. In all, the priority effects observed in the *ex situ* S-bead recolonization experiments highlight the importance of initial community composition in determining subsequent assembly patterns. Genome-resolved analyses revealed that these taxonomic shifts were accompanied by pronounced genomovar-level variation within dominant taxa, reflecting ecotype differentiation and microhabitat-specific variant turnover. This genomic stratification is consistent with microhabitat filtering acting within dominant lineages, and suggests selection among coexisting variants across attached versus planktonic fractions. Together, the concordant ASV- and MAG-level patterns support a model in which historical contingency (priority effects) interacts with deterministic substrate- and microhabitat-imposed filters to structure sulfur-associated communities. These findings indicate that microbial succession is influenced not only by species turnover but also by intraspecific genomic structure, highlighting that community assembly operates simultaneously at inter- and intra-species scales.

Both *in situ* and *ex situ* experiments provided novel insights into the ecological processes governing microbial community dynamics in acidic environments and the contribution of stochastic and deterministic forces. Free-living microbial communities were structured by a balance between stochastic drift and deterministic selection, with flow-dependent effects influencing dispersal limitation. Free-living communities under high-flow conditions exhibited stronger signatures of deterministic selection compared to low-flow conditions, likely due to greater environmental filtering associated with turbulent flow and shorter residence times. In contrast, low-flow communities exhibited a greater contribution of stochastic processes, suggesting that in less turbulent conditions, microbial community assembly is largely influenced by random colonization events rather than selective pressures.

Particle-associated microbial communities, regardless of flow conditions, exhibited a strong stochastic signature, with drift contributing 63%–67% of community turnover. This suggests that the continuous deposition and resuspension of particles from multiple sources (e.g. volcanic inputs, riverbank inputs, and sediment resuspension) promote a dynamic microbial assemblage where random processes play a major role. In contrast, S-bead associated communities exhibited strong deterministic assembly patterns, with homogeneous selection contributing 57.2% of community structuring. This shift from stochastic to deterministic influence suggests that stable colonization surfaces impose consistent selective pressures that override background environmental variability. Thus, the transition from free-living to a surface-attached biofilm represents an ecological filter, favoring specific traits such as surface adhesion, biofilm formation, and sulfur metabolism. These ecological patterns were reflected in the community’s metabolic potential. Free-living fractions retained broad metabolic versatility, emphasizing growth, motility, and biosynthesis, consistent with a dispersal-focused lifestyle. Particle-associated communities were enriched in environmental sensing and ion metabolism, reflecting adaptation to heterogeneous surfaces. S-beads favored substrate acquisition, energy conversion, and signal transduction, highlighting specialization for surface attachment. Sequential recolonization led to further functional narrowing, with a reduced set of specialized taxa dominating the community and allocating a greater proportion of their genomes to surface-associated functions. This was accompanied by a loss of overall metabolic redundancy. Observed functional shift support a transition from broadly versatile colonizers to streamlined, niche-adapted consortia during recolonization, consistent with strong priority effects, ecotype selection, and substrate-driven filtering in the assembly of sulfur-attached microbiomes. This trend is also consistent with reduced functional redundancy under microcosm transfer, where repeated bottlenecks and competitive sorting can amplify deterministic filters.

## Conclusions

This study provides novel insights into microbial community assembly and succession in extreme acidic environments by integrating spatial partitioning, *in situ* colonization, and *ex situ* recolonization approaches. We demonstrated that water flow, particle association, and sulfur surface colonization shape microbial succession and assembly, with free-living communities exhibiting higher richness and temporal variability than particle-associated microbiomes. The structured successional trajectory observed in S-bead colonization highlights the foundational role of sulfur-oxidizing taxa in biofilm formation and subsequent microbial recruitment. Additionally, strong priority effects in *ex situ* recolonizations suggest that early colonizers exert long-term influence over community composition, and highlight the importance of colonization history in determining community trajectories. Our findings underscore the importance of both deterministic and stochastic processes in structuring microbial diversity in acidic riverine systems, with flow-dependent selection influencing free-living communities, drift dominating particle-associated communities, and stable colonization surfaces promoting increased effect of selection in biofilm-associated communities. By elucidating these dynamics, this study provides a framework for better understanding microbial ecology in these extreme ecosystems and offers insights relevant to biotechnological applications of extreme acidophiles and their communities.

## Supplementary Material

Supplementary_material_wrag048

## Data Availability

Metagenomic sequencing data have been deposited in the NCBI Sequence Read Archive (SRA) under BioProject accession ID PRJNA914835. Amplicon sequencing data and recovered MAGs are available under the same BioProject. Sample metadata and associated accession numbers are summarized in Table S1.
