# Supplementary material for: Microbial succession and assembly shaped by sulfur, spatial partitioning, and water flow in a volcanic acidic river of northern Patagonia
Source: ISME J. 2026 Mar 9;20(1):wrag048. doi: 10.1093/ismejo/wrag048 (PMC13122624; doi:10.1093/ismejo/wrag048)
Supplement: Supplementary_material_wrag048 [file supplementary_material_wrag048.zip › Supplementary_information.docx]

**Microbial succession and assembly shaped by sulfur, spatial partitioning, and water flow in a volcanic acidic river of northern Patagonia.**

Duarte-Ramírez Juan^1,2^†, Arisan Dilanaz^1,2^†, Rojas-Villalobos Camila^1,3^, Díaz-González Fernando^1,2^, Sepúlveda-Rebolledo Pedro^1^, Moya-Beltrán Ana^4^, Ulloa Ricardo ^5^, Johnson D. Barrie^6,7,8^, Vera Mario^9,10^, Beatriz Díez,^1,11,12^, Castro Matías^13^, Temporetti Pedro^14^, Giaveno Alejandra^5^, Issotta Francisco^1,11^, Quatrini Raquel^1,15^*

1 Centro Científico y Tecnológico de Excelencia Ciencia & Vida, Santiago, Chile

2 Programa de Doctorado en Biotecnología y Bioemprendimiento, Facultad de Medicina, Universidad San Sebastián, Santiago, Chile

3 Programa de Doctorado en Biología Computacional, Facultad de Ingeniería, Arquitectura y Diseño, Universidad San Sebastián, Santiago, Chile

4 Departamento de Informática y Computación, Facultad de Ingeniería, Universidad Tecnológica Metropolitana, Santiago, Chile

5 PROBIEN (CCT Patagonia Confluencia-CONICET, UNCo), Facultad de Ingeniería, Departamento de Química, Universidad Nacional del Comahue, Neuquén, Argentina

6 School of Biological Sciences, Bangor University, Bangor LL57 2UW, United Kingdom

7 Faculty of Health and Life Sciences, Coventry University, Coventry, United Kingdom

8 Natural History Museum, London, United Kingdom

9 Instituto de Ingeniería Biológica y Médica, Escuelas de Ingeniería, Medicina y Ciencias Biológicas, Pontificia Universidad Católica de Chile, Macul, Santiago 7820436, Chile.

10 Departamento de Ingeniería de Minería, Escuela de Ingeniería, Pontificia Universidad Católica de Chile, Macul, Santiago 7820436, Chile.

11 Centro GEMA - Genómica, Ecología & Medio Ambiente, Universidad Mayor, Chile.

12 Millennium Institute Center for Genome Regulation (CGR), Santiago, Chile.

13 Instituto Milenio de Oceanografía (IMO), Universidad de Concepción, Concepción, Chile

14 Instituto de Investigaciones en Biodiversidad y Medioambiente (INIBIOMA), Centro Regional Universitario Bariloche-UNComahue, CCT-Patagonia Norte, CONICET, San Carlos de Bariloche, Argentina

15 Facultad de Ciencias, Universidad San Sebastián, Santiago, Chile

† Join First Authors

* Correspondence

Raquel Quatrini

[rquatrini@cienciavida.org](mailto:rquatrini@cienciavida.org); raquel.quatrini@uss.cl

Centro Ciencia & Vida

Facultad de Ciencias, Universidad San Sebastián

Ave. Del Valle Norte 725, Huechuraba

Santiago, Chile Avenida del Valle Norte 725

8580702 Huechuraba

Santiago

Chile

**Additional information**

Additional information supporting and expanding the results presented in this study are provided as Supplementary Tables (8 display items) and Supplementary Figures (6 display items). Detailed legends are presented below and included in each display item.

**Table S1. Description of samples, experiments and treatments used in this study. (a)** Sample names correspond to their respective experimental conditions and treatments. All samples listed were collected from Rio Agrio Superior at Cascada de la Culebra (RAS-CC, -37.885454 S, -71.067138 W) between 2019 and 2023. Native water samples were collected at the plunge-pool of RAS-CC from high-flow (H, 0.1 m/s) and low-flow (L, 0.03 m/s) stream sectors. Water was subjected to sequential filtering through 8 µm pore filters (P, particles) and 0.22 µm pore filters (F, filtrate). The *in situ* colonization samples represent sulfur beads (S-beads) exposed in the water column for different time intervals (24–96 hours) under low-flow conditions, with microbial communities collected from the attached fraction. The *ex situ* recolonization samples correspond to the first (R1) and third phase (R3) of recolonization experiments conducted under controlled conditions, using previously colonized S-beads as inoculum as indicated. Environmental parameters, including sampling day and time, temperature, and pH, were recorded for each sample. **(b)** Summary of 16S rRNA gene amplicon sequencing depth and read-quality metrics across sample groups. For each sample group (as defined in **Table S1a**), triplicate-averaged Phred quality scores (minimum, mean, and maximum across reads after processing), the total number of retained sequences (Total Seqs), the target read length (Seq Length; bp). **(c)** Sequencing depth, assembly statistics, and functional annotation summary for shotgun metagenomes from RAS-CC in situ microhabitats and ex situ recolonization experiments. For each shotgun metagenome, sequencing yield (number of reads and bases), assembly statistics (number of contigs, total assembled length, longest contig, N50 and N90; minimum contig length = 200 bp), and estimated library complexity (Nonpareil effective diversity) are reported. It also summarizes contig-level taxonomic assignment counts across ranks (superkingdom to species) including assignment congruence/disparity metrics, gene prediction features (ORFs, rRNAs, tRNAs/tmRNAs), functional annotation totals (KEGG, COG, Pfam), and binning outcomes and quality summaries (completeness/contamination thresholds). Sample codes correspond to the microhabitat/fraction and timepoint definitions and identifiers in Table S1a.

**Table S2.** **Physicochemical characteristics of native water column samples at Rio Agrio, Cascada de la Culebra (RAS-CC)**. Physicochemical parameters were recorded *in situ* during sample collection at RAS-CC using a multiparametric probe (Hanna HI9829). Measured parameters included: geographic coordinates, date and time of sampling, pH, temperature (Temp, ºC), atmospheric pressure (Press, PSI), oxidation-reduction potential (ORP, mV), dissolved oxygen (DO, ppm), electroconductivity (EC, µS/cm), resistance (RES, Ohm-cm), salinity (Sal, PSU), total dissolved solids (TDS, ppm) , and turbidity (Turb, FNU). Elemental composition of the water column was analysed *ex situ* by inductively coupled plasma mass spectrometry (ICP-OES, Perkin Elmer 7300 DV) following EPA standard 200.7 (U.S. EPA, 1994), quantifying concentrations (ppm) of Magnesium (Mg), Calcium (Ca), Iron (Fe), Sodium (Na), Aluminium (Al), Potassium (K), Manganese (Mn), Strontium (Sr), Zinc (Zn), Lithium (Li), Copper (Cu), Vanadium (V), and Nickel (Ni). Sampling campaigns were conducted between 2019 and 2023, and included yearly periodic sampling and diel cycle monitoring at 8, 15, and 20 h under high-flow and low-flow conditions (2023 only). Averaged values listed correspond to multiple measurements collected across sampling periods.

**Table S3. Overview of microbial community diversity metrics across samples and experiments.** Key microbial diversity indices were calculated using QIIME2 v2021.8. (**a**) Summary of computed alpha diversity metrics categorized into five main groups: (i) richness, (ii) overall diversity, (iii) evenness, (iv) dominance, and (v) specialized indices. Descriptive statistics for these metrics, including mean and standard deviation, are provided. (**b**) ANOVA results for diversity and richness metrics across native samples recovered from the water column at RAS-CC. Factors: fraction, time, and flow. (**c**) ANOVA results for diversity and richness metrics in the *ex situ* recolonization experiment. (**d**) Summary of Tukey's post-hoc tests for pairwise comparisons across diversity metrics in the *ex situ* recolonization experiment. Pairwise comparisons of microbial diversity indices across recolonization cycles to identify significant differences between conditions.

**Table S4.** **Relative abundance of the microbial taxa in the native RAS-CC water column samples.** Taxonomy (up to species-level rank) assigned against the **Greengenes2 16S rRNA database** is indicated. Only taxa with a relative abundance per sample above 0.01% were retained for further analysis. Abundance was ranked as high (H, > 1%), medium (M, <1% and > 0.1%) or low (< 0.1% and >0.01%) and data coloured accordingly. The dataset includes the ASV identifier, taxonomy, classifiers, presence/absence across samples (occurrence), relative abundance per sample (%) and rank abundance per sample (H, M, L). Sample designation is that shown in **Table S1**. Occurrence frequencies are reported as absolute counts and percentages, with thresholds defined as presence in ≥50% of fraction replicates. **Abbreviations**: WF, water filtrate; WP, water particles; rank abundance: H, high; M, medium; L, low.

**Table S5.** **Relative abundance of the microbial taxa in *in situ* colonized S-beads.** Taxonomy (up to species-level rank) assigned against the **Greengenes2 16S rRNA database 2022.10** is indicated. Only taxa with a relative abundance per sample above 0.01% were retained for further analysis. Abundance was ranked as high (H, > 1%), medium (M, <1% and > 0.1%) or low (< 0.1% and >0.01%) and data coloured accordingly.

The dataset includes the ASV identifier, taxonomy, classifiers, presence/absence across samples (occurrence), relative abundance per sample (%) and rank abundance per sample (H, M, L). Sample designation is that shown in **Table S1**. Occurrence frequencies are reported as absolute counts and percentages, with thresholds defined as presence in ≥50% of fraction replicates. **Abbreviations**: C, *in situ* colonization; SB, sulfur beads, timeline: E, early (0-24h); M, mid (48-72h); L, late (96h); rank abundance: H, high; M, medium; L, low.

**Table S6.** **Relative abundance of the microbial taxa in *ex situ* colonized S-beads.** Taxonomy (up to species-level rank) assigned against the **Greengenes2 16S rRNA database 2022.10** is indicated. Taxa with a relative abundance per sample above 0.01% were retained for further analysis. Microbial abundance was ranked as high (H, > 1%), medium (M, 0.1%-1%) or low (0.01%-0.1%) and data coloured accordingly. The dataset includes the ASV identifier, taxonomy, classifiers, presence/absence across samples (occurrence), relative abundance per sample (%) and rank abundance per sample (H, M, L). Sample designation is that shown in **Table S1**. Occurrence frequencies are reported as absolute counts and percentages, with thresholds defined as presence in ≥50% of fraction replicates. **Abbreviations**: C, *in situ* colonization; SB, sulfur beads, timeline: R1, recolonization cycle 1; R3, recolonization cycle 3; AF, attached fraction; PF, planktonic fraction; 24, 24h; 48, 48h; rank abundance: H, high; M, medium; L, low.

**Table S7. Summary of ASV dataset complexity across experiments.** Overview of sequencing output and dominance structure for the three experimental datasets analyzed in this study: in situ water-column communities, in situ sulfur-bead (S-bead) colonization communities, and ex situ S-bead recolonization communities. The subset of ASVs retained for downstream analyses was defined as ASVs present in >50% of samples within each experiment. The proportion of retained ASVs is expressed as a percentage of the total ASV pool. Community dominance is summarized as the cumulative relative abundance explained by the top 10, top 3, and top 1 most abundant retained ASVs, respectively. References to the corresponding full taxonomic and abundance tables are provided (Tables S4–S6).

**Table S8. PERMANOVA on Bray–Curtis dissimilarities of microbial community profiles during ex situ S-bead recolonization experiments.** PERMANOVA results based on Bray–Curtis dissimilarities of ASV relative-abundance profiles from the ex situ S-bead recolonization experiment. Pairwise comparisons test the effects of fraction (attached vs planktonic), inoculum stage (24 h vs 48 h), and transfer history (R0 vs R1 vs R3). The table reports F statistics (F), variance explained (R²), permutation p-values (P), and Benjamini–Hochberg adjusted p-values (P_adj_BH).

**Table S9. Metadata and assembly statistics of MAGs recovered from RAS-CC colonization experiments.** Summary of genome-resolved metagenomic data for the 44 dereplicated MAGs obtained across RAS-CC microhabitats. The table includes sample identifiers, project, organism, and sample metadata, as well as assembly metrics.

**Table S10. Relative importance of ecological processes across microhabitats.** Summary statistics are provided for each ecological process, Dispersal Limitation, Homogeneous Dispersal, Drift (Others), Heterogeneous Selection, and Homogeneous Selection, across five RAS-CC microhabitats: Filtrate, Particles and S-beads. For each process and habitat, the number of samples (n), mean relative importance (%), standard deviation (sd), and standard error (se) are reported. Statistical differences in the relative importance of each process were evaluated using two-sided Wilcoxon rank-sum tests, with p-values adjusted using the Bonferroni correction method.

**Supplementary Figures**

**Figure S1. Experimental design for *in situ* colonization of S-beads**

(**A**) Schematic representation of flow-through columns used for *in situ* colonization of S-beads at RAS-CC. Each column (**I**) was packed with sterile quartz (as inert support) and S-beads (20 g each) of similar granulometry (3-4 mm) and secured within a modified test‑tube rack used as a support frame, then submerged 15 cm below the air-water interface in a low-flow region of the plunge pool, aligned with the water flow. The open design (**II**) allowed continuous water exchange, facilitating microbial attachment to the sulfur beads. Four independent columns were deployed and retrieved at 24, 48, 72, and 96 h. Upon retrieval (**III**), S-beads were collected with sterile tweezers, transferred into sterile falcon tubes containing site-filtered water, and stored at 4°C until further processing. Once in the laboratory, 10 g of S-beads were washed with acidified water to remove loosely-attached cell. Cells were detached by gentle inversion in 0.05% Triton X-100, counted, and filtered through 0.22 µm polyethersulfone membrane disc filters (Millipore) for DNA extraction. The extracted DNA was used for both targeted metagenomic sequencing (16S rRNA) and whole-metagenome sequencing (**Table.S1**). Water flow measurements were conducted before deployment to ensure placement in a low-flow zone, and environmental parameters (temperature and pH) were monitored throughout the experiment. (**B**) Relationship between bacterial cell counts and ASV numbers during in situ colonization. **I.** The graph depicts total bacterial cell counts (lines) alongside the number of assembled ASVs (bars) at each time point (24, 48, 72, and 96 h). Both free-living cells fractions recovered from the RAS-CC water column (green) and the S-beads attached cells (red) are shown. Cell counts (in triplicate) revealed an average water column microbial concentration of ~10x cells/mL (4 days average). S-beads exhibited significantly higher cell counts compared to the water, with initial attachment observed at 10⁷ cells/gr of sulfur after 24h. During in situ colonization of the S-beads, cell counts increased steadily, peaking at 1.68 × 10⁸ cells/gr of sulfur after 72h, and declined slightly after 96h at the site. Sequencing of the S-beads attached microbiomes generated a total of 29,835 ASVs across timepoints, with 514 ASVs remaining after removal of singletons (**Table S5**). With or without singletons, trend described for cell counts, was mirrored for ASVs. Total ASV counts (yellow) and ASVs excluding singletons (purple) per time point are depicted. **II.** During the experiment, the water pH remained relatively stable and acidic, whereas the water showed a thermal variation of ±10 °C.

**Figure S2. Experimental design for *ex situ* colonization of S-beads**

(**A**) Schematic representation of the *ex situ* recolonization experiment for S-beads. Recolonization assays were conducted under controlled conditions using minimal saline medium with trace elements (MSM+TE, pH 2.5). *In situ* colonized sulfur beads (S-beads) retrieved at 24 h and 48 h from RAS-CC were used as the initial inoculum (R0). Two microbial fractions were derived from R0: (I) an attached fraction (AF) consisting of adherent biofilm-forming microbial cells and (II) a planktonic fraction (PF) containing detached or dispersed cells from the surrounding supernatant. These fractions served as inoculant in three consecutive recolonization cycles (R1, R2, R3), each lasting 3 days. (**B**) Representation of microbial recolonization dynamics across three sequential cycles. (**I**) First Recolonization (R1): S-beads previously exposed at RAS-CC during the *in situ* colonization experiment, and harvested at 24 h (C-SB-24) and 48 h (C-SB-48), were mixed with sterile S-beads and incubated in MSM+TE for three days. After incubation, microbial cells from the beads were harvested for DNA extraction (R1-AF-24; R1-AF-48). Also, sterile S-beads (5% w/v) were exposed to the planktonic fraction (PF) in which the *in situ* colonized beads had been stored in a 1:1 ratio (v/v) with MSM+TE to evaluate recolonization from planktonic communities (R1-PF-24, R1-PF-48). (II) Second Recolonization (R2): Microbial communities from R1 were transferred onto fresh sterile S-beads (1:1 ratio), and recolonization was allowed to proceed under identical conditions (R2-AF-24; R2-AF-48, R2-PF-24, R2-PF-48). (III) Third Recolonization (R3): Sulfur beads from R2 were used as the inoculum for the final recolonization phase. Microbial communities were again transferred onto fresh sterile S-beads (1:1 ratio) and incubated under batch culture conditions (R3-AF-24; R3-AF-48, R3-PF-24, R3-PF-48). Each recolonization cycle was performed at 30°C under batch culture conditions After incubation, S-beads attached microbial fractions were collected for genomic DNA extraction and sequencing (R1 and R3 were used for amplicon sequencing; R2 metagenomic sequencing). **Abbreviations:** AF (attached fraction), PF (planktonic fraction), R (recolonization cycle).

**Figure S3. Taxonomy and partition of rare microbial taxa between particle-associated and free-living communities at RAS-CC.** (**A**) Dot plot of the count of low-abundance ASVs per phylum (0.01 - 0.1% abundance), categorized by their presence in the filtrate and/or particle fractions. (**B, C**) Back-to-back bar chart depicting the cumulative ASVs abundance per phylum in both filtrate and particle fractions (**B**), or exclusively in either fraction (**C**). This figure complements Figure 4 by focusing on highly diverse low-abundance ASVs (0.01–0.1% abundance).

**Figure S4. Microbial community clustering across RAS-CC fractions and colonization treatments**

Clustering of ASVs based on Bray-Curtis dissimilarity distance per RAS-CC water column fraction (filtrate, particles) and per colonization experiment (*in situ* colonization and *ex situ* recolonization cycles R1 and R3). Additional details on the beta diversity analysis can be found in **Fig. S5 and Table S8**.

**Figure S5. Beta diversity analysis of microbial communities from RAS-CC and** *ex situ* **experiments**(**A**) Heatmap and dendrogram representation, with a scale from 0 to 1, where 1 (red) indicates maximum dissimilarity (samples have highly distinct taxonomic compositions), and 0 (blue) represents samples with similar or identical taxonomic compositions. (**B**) Non-Metric Multidimensional Scaling (NMDS) of ASV samples using Bray-Curtis beta diversity. Statistical analysis: PERMANOVA (R² = 0.99664; F = 4890.7; p < 0.05).

**Figure S6**. **COG pathway enrichment analysis across microhabitats.** Pairwise comparisons of gene functional enrichment across microhabitats were conducted based on the proportion of genes assigned to COG pathways. The left panels show the proportion of genes with COG assignment in the metagenomes of the respective samples. The central panels display the proportional mean differences, along with 95% confidence intervals. Statistically significant differences (p < 0.05, corrected using the Benjamini-Hochberg method) are highlighted in the colour of the sample in which the category is enriched. Genes with a minimum length of 100 amino acids and a TPM value >1 were included in the analysis. The right panels show the log2-fold TPM change of genes with COG assignment in the same set of metagenomes. (**A**) Functional differences between particle-associated and free-living communities. Genes related to signal transduction mechanisms and inorganic ion transport/metabolism were significantly enriched in particle-associated communities. In contrast, free-living communities exhibited a higher proportion of genes involved in translation and ribosomal functions, suggesting increased protein synthesis activity. **(B**) Functional enrichment in S-beads associated communities. Compared to free-living bacteria, the sulfur bead-associated community showed a higher representation of genes involved in energy production, amino acid transport and metabolism, carbohydrate transport, and signal transduction. This suggests that S-beads surfaces provide a stable and energy-rich microhabitat, promoting microbial colonization and metabolic specialization. (**C**) Functional specialization in communities assembled after sequential recolonization. Recolonized microbial communities displayed enrichment in genes related to cell envelope biogenesis, carbohydrate metabolism, cell motility, secretion, and secondary metabolite production. These patterns suggest a selective process where successful colonizers maintain and enhance traits essential for surface attachment, dispersal, and competition within sulfur-rich environments.
