# Supplementary figures and images for: Microbial succession and assembly shaped by sulfur, spatial partitioning, and water flow in a volcanic acidic river of northern Patagonia
Source: ISME J. 2026 Mar 9;20(1):wrag048. doi: 10.1093/ismejo/wrag048 (PMC13122624; doi:10.1093/ismejo/wrag048)

A

I.

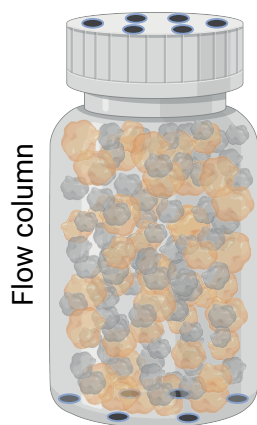

- S-beads 20 gr  
 Quartz 20 gr

II.

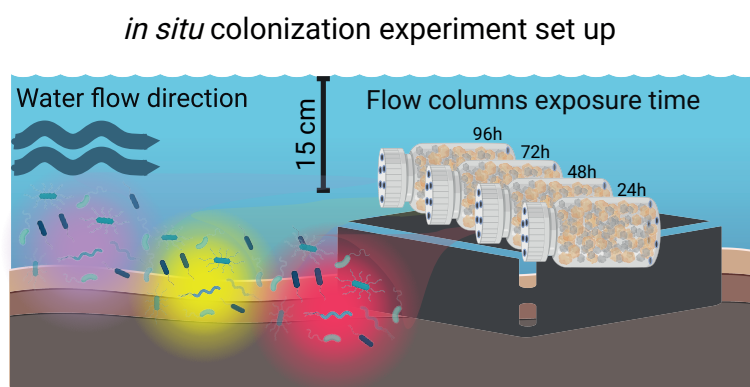

III.

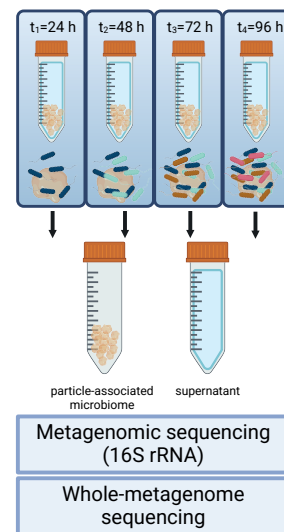

B

I.

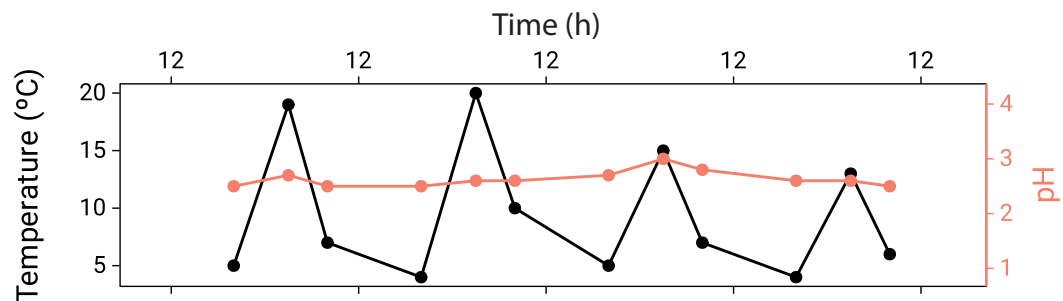

II.

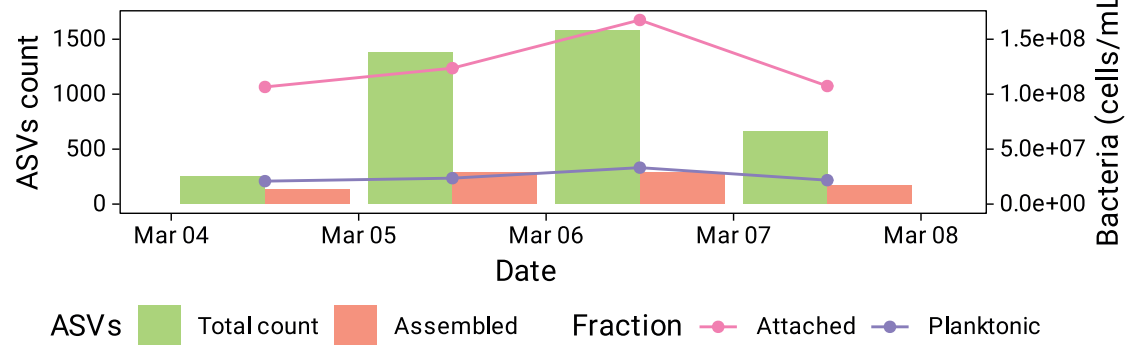

Supplement: Supplementary_material_wrag048 [file supplementary_material_wrag048.zip › FigS1.pdf]

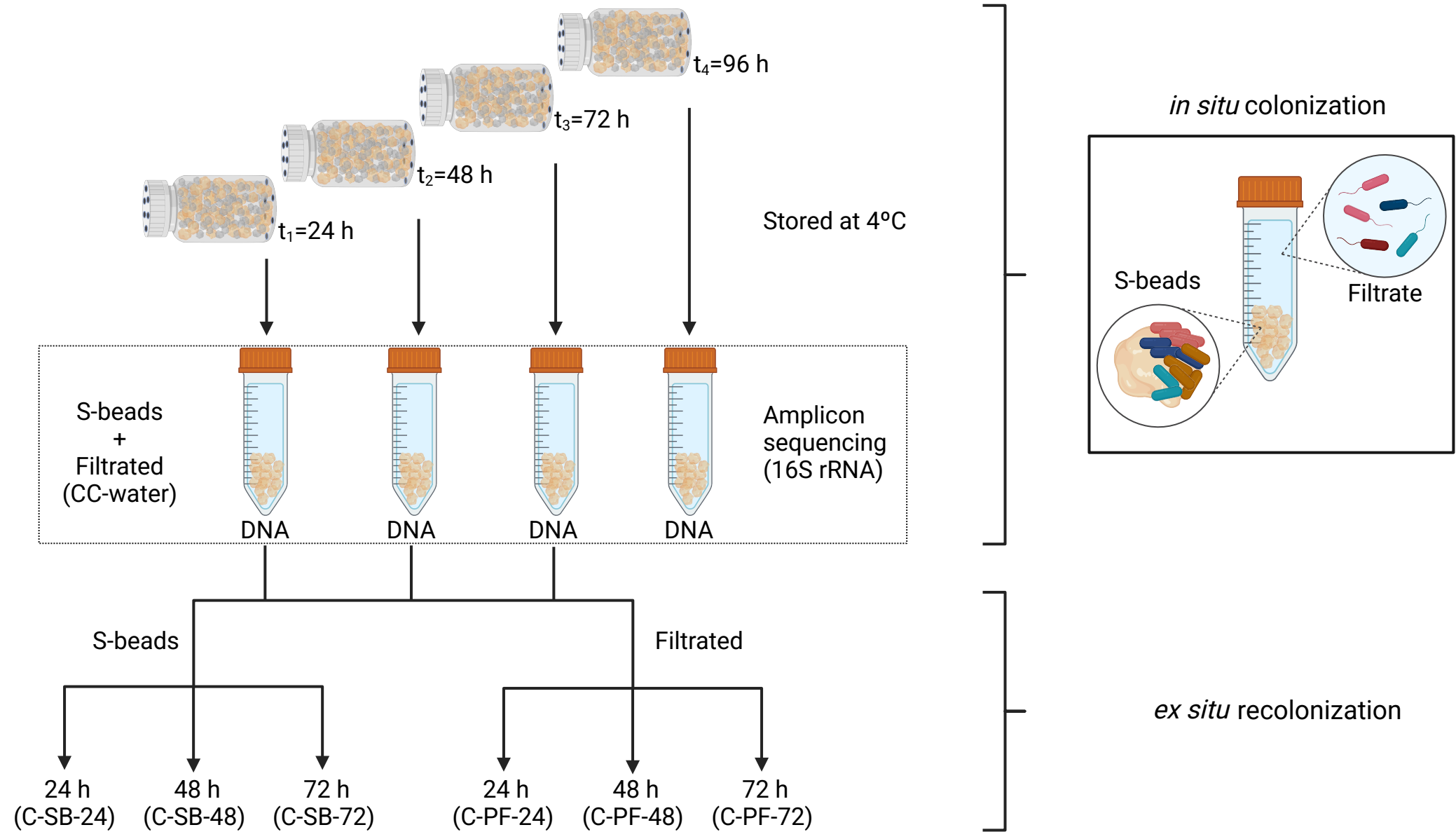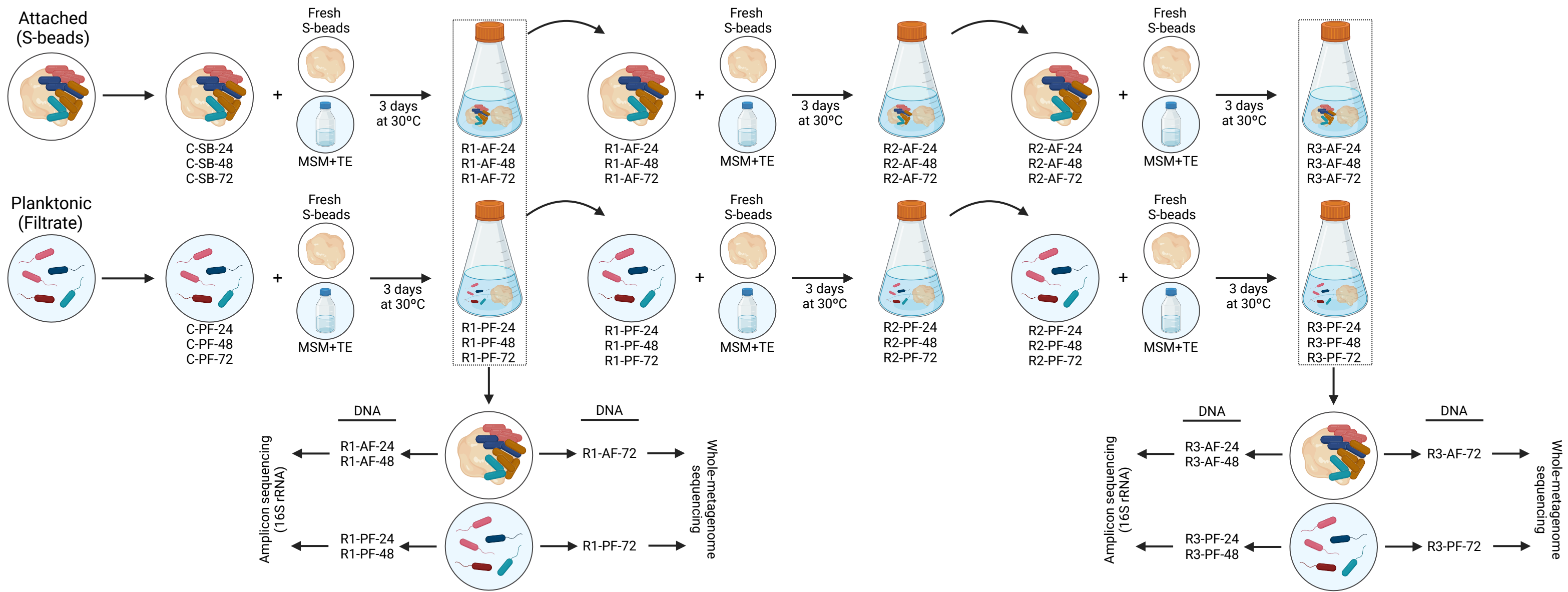

Supplement: Supplementary_material_wrag048 [file supplementary_material_wrag048.zip › FigS2.pdf]

A

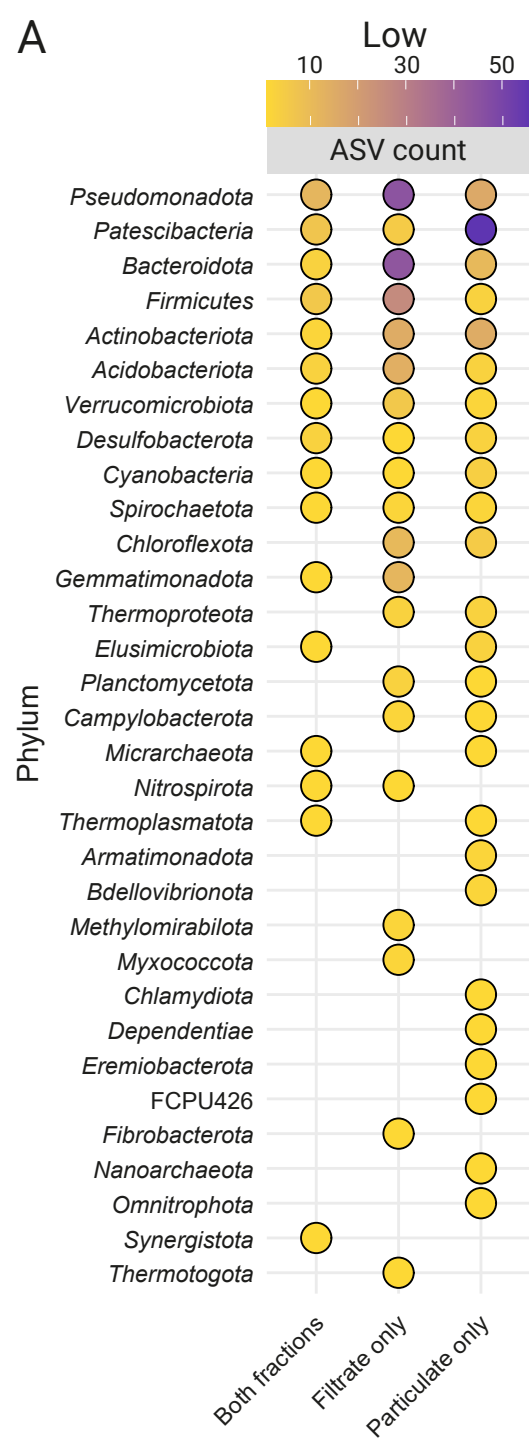

B

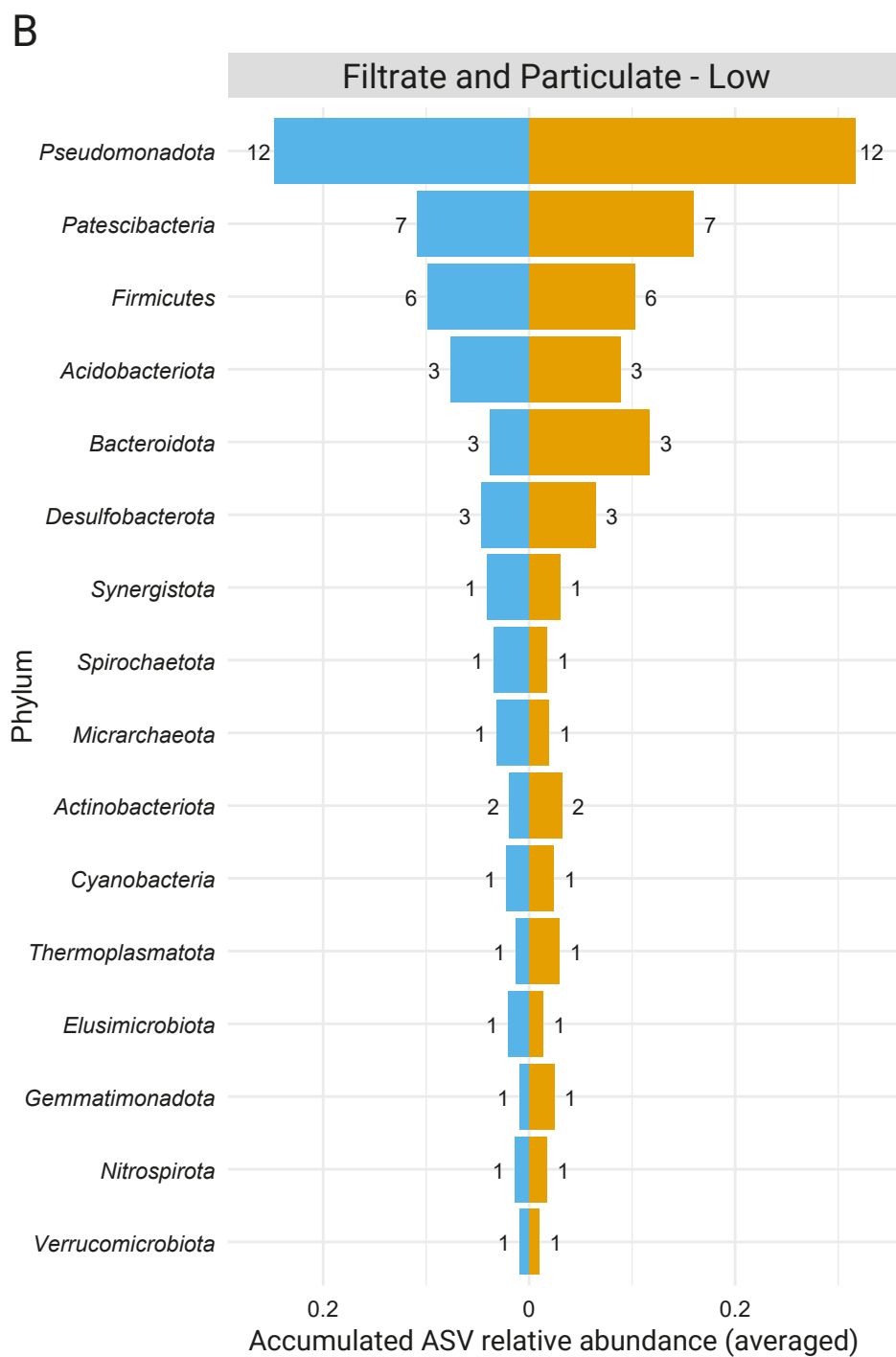

C

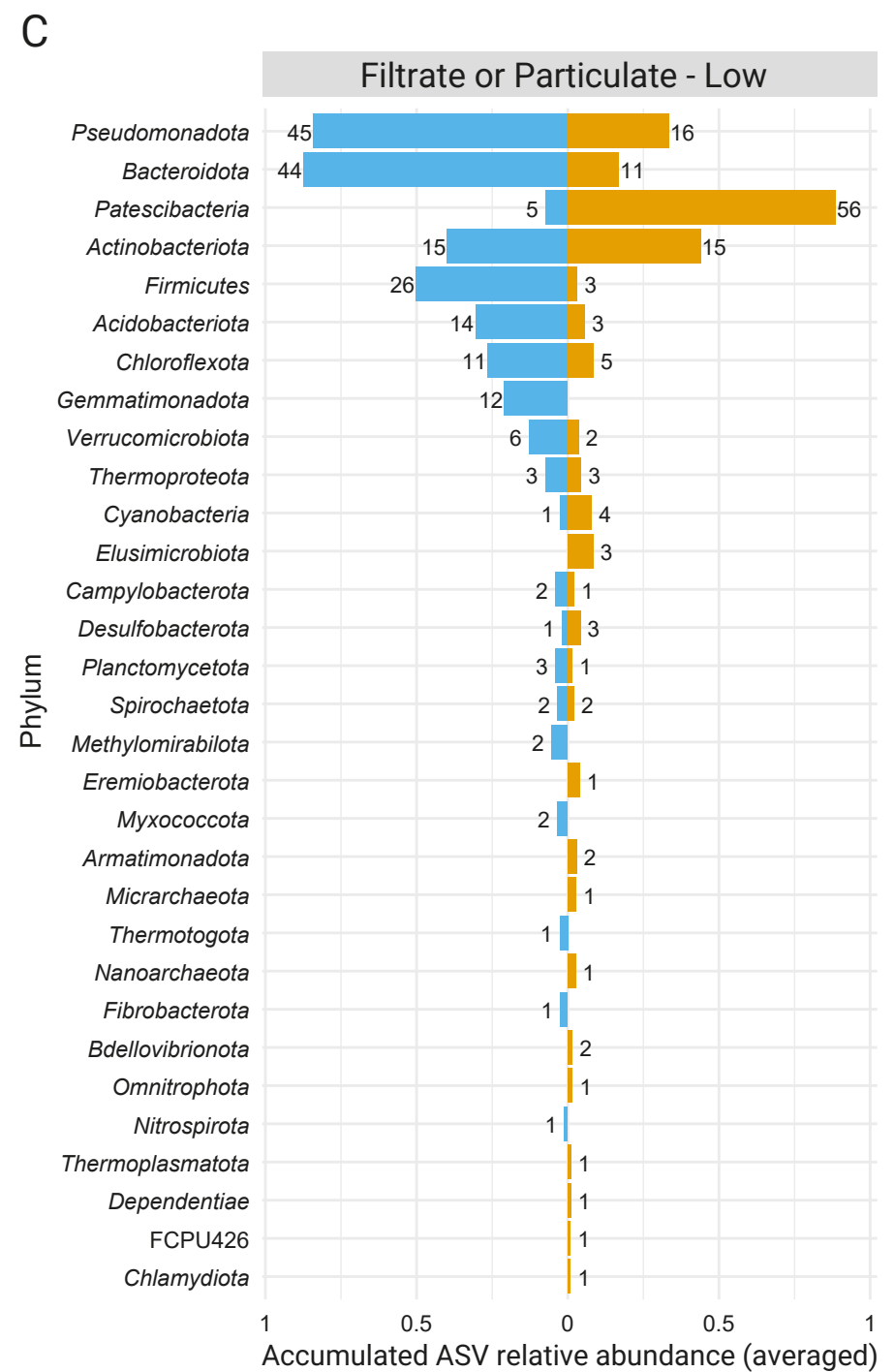

Supplement: Supplementary_material_wrag048 [file supplementary_material_wrag048.zip › FigS3.pdf]

Abundance

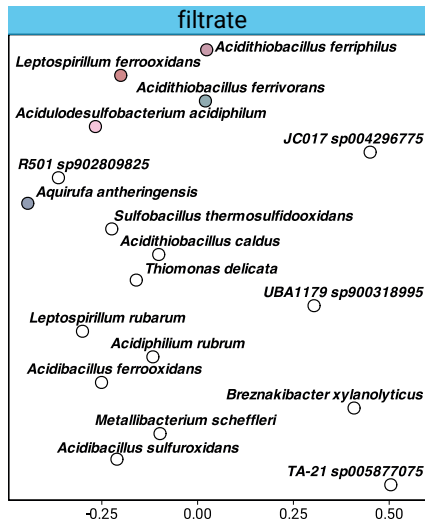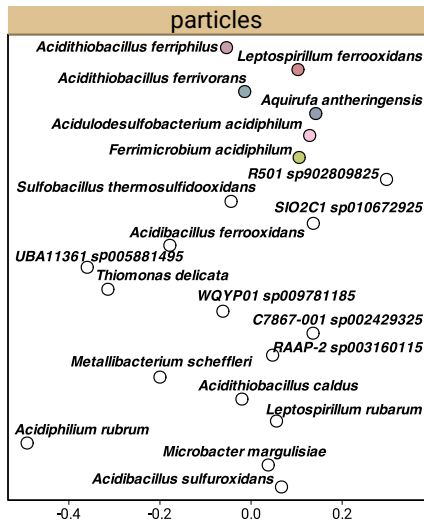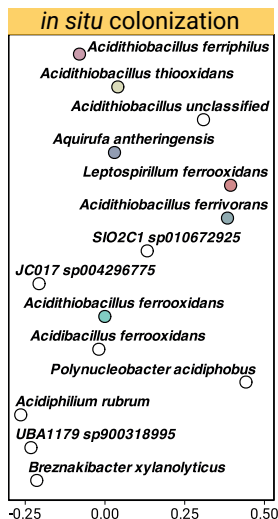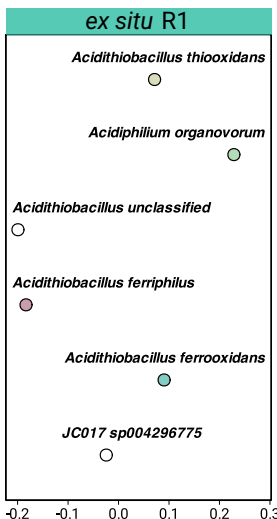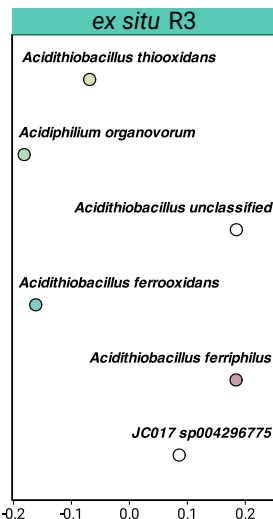

Bray-Curtis distance

Supplement: Supplementary_material_wrag048 [file supplementary_material_wrag048.zip › FigS4.pdf]

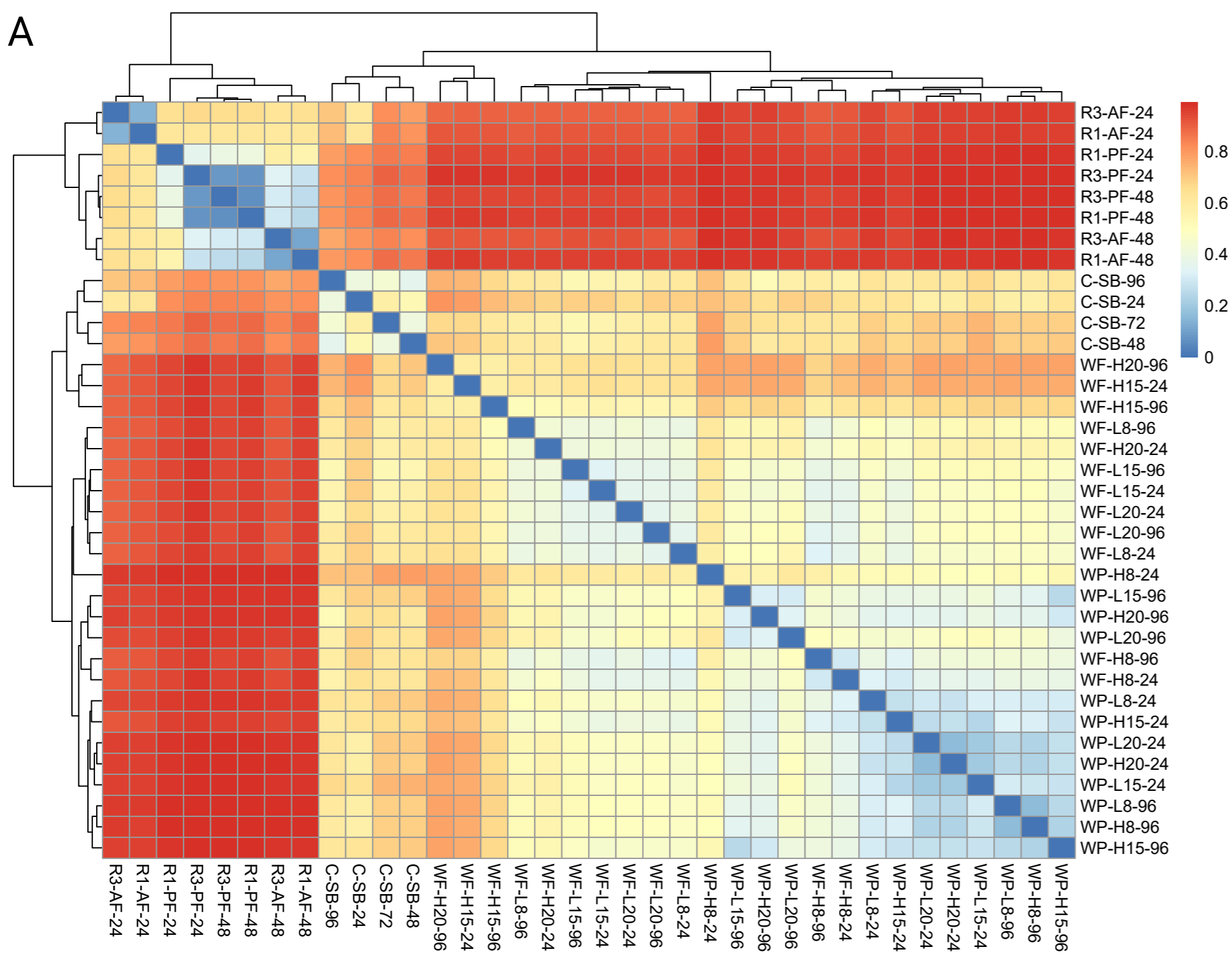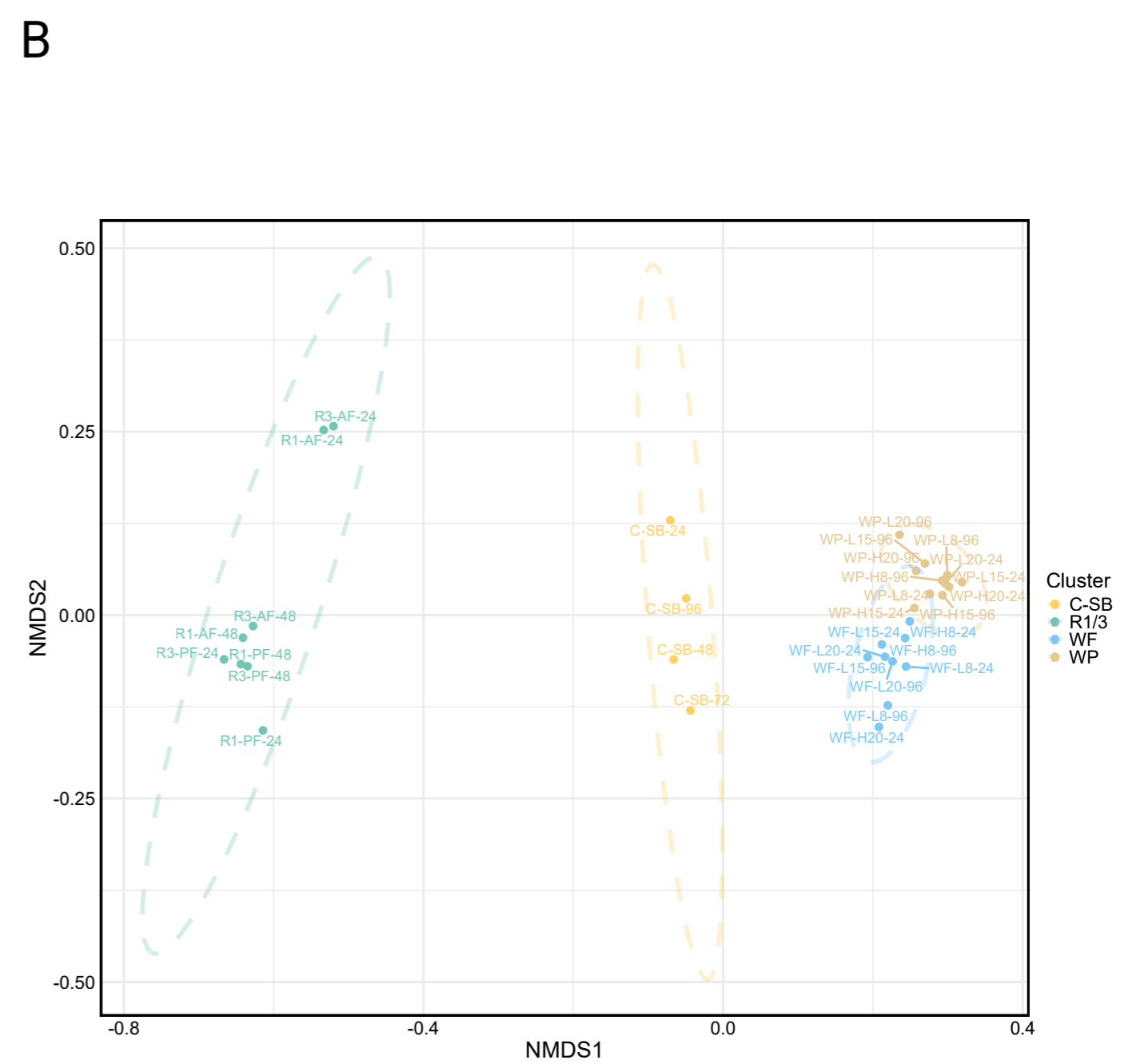

Supplement: Supplementary_material_wrag048 [file supplementary_material_wrag048.zip › FigS5.pdf]

A

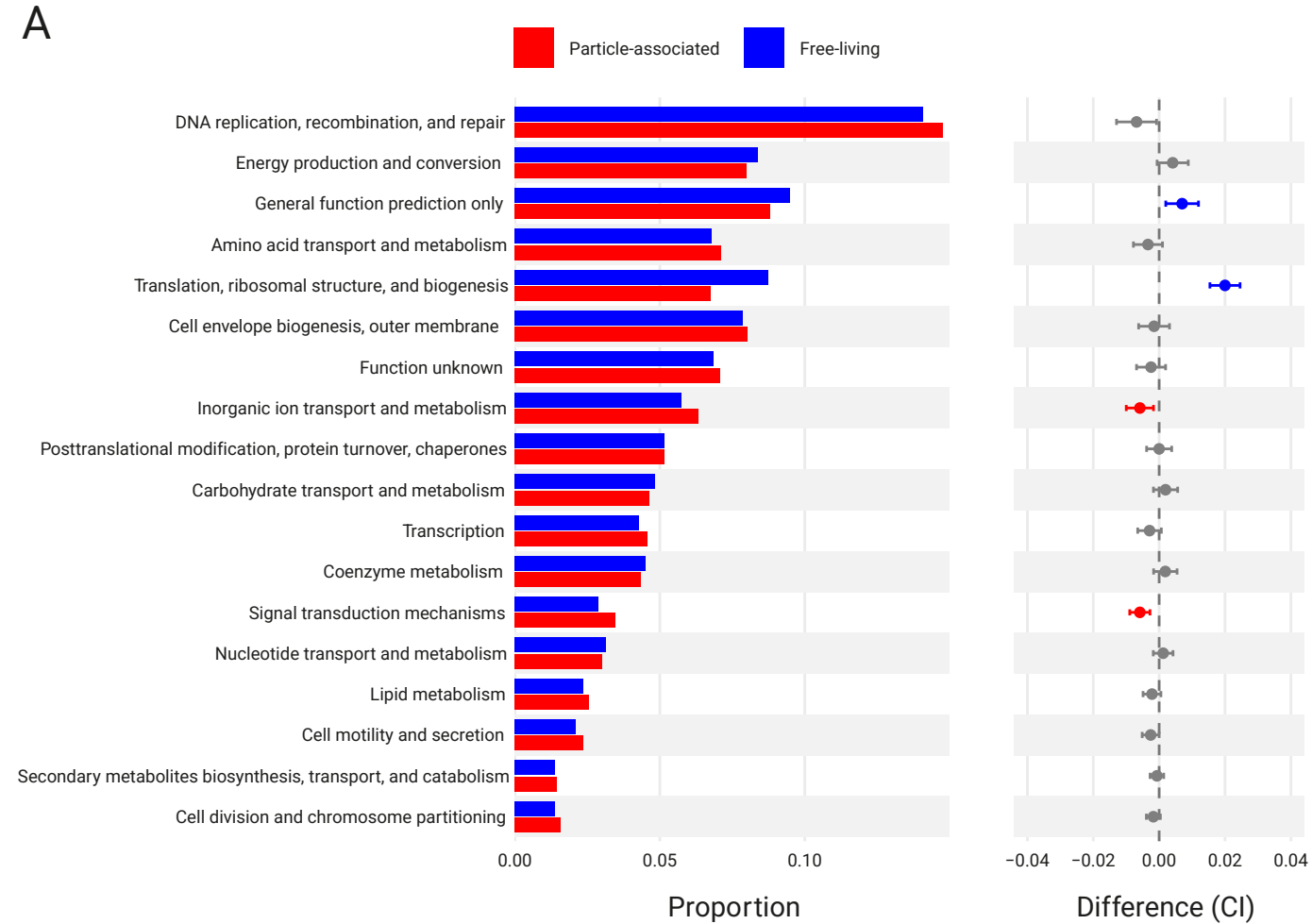

B

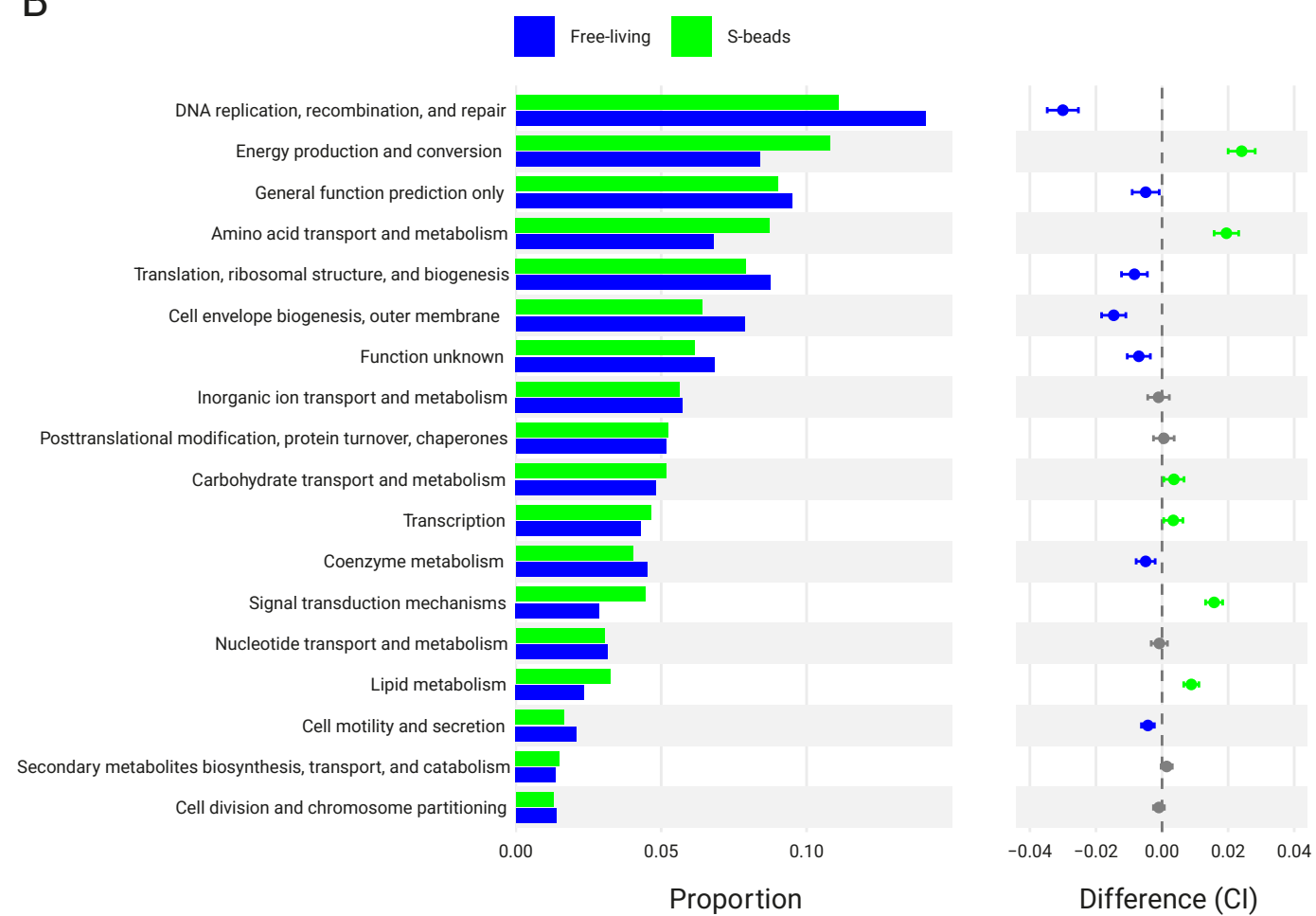

C

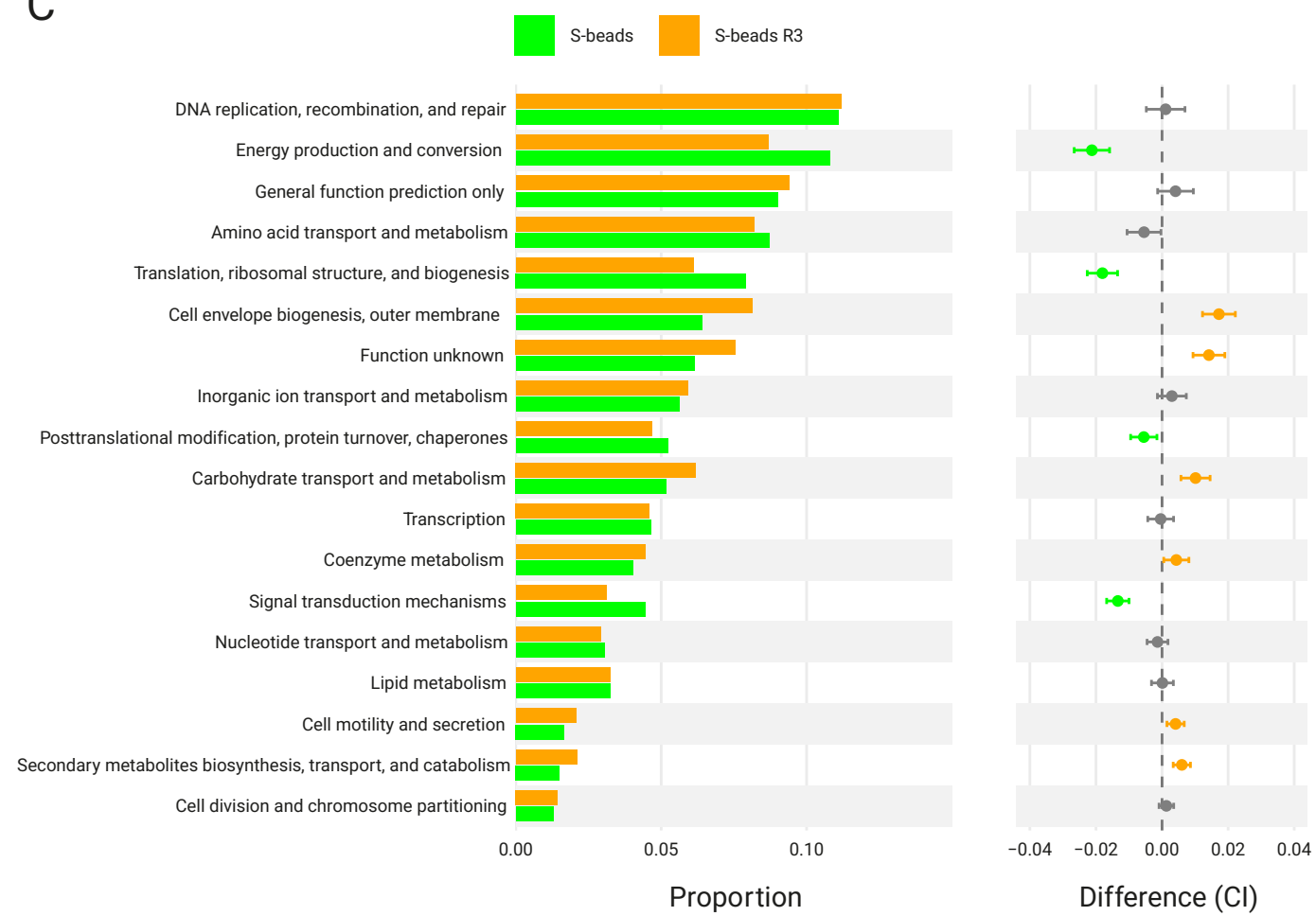

Supplement: Supplementary_material_wrag048 [file supplementary_material_wrag048.zip › FigS6.pdf]
